# How Does Solvation Layer Mobility Affect Protein Structural Dynamics?

**DOI:** 10.3389/fmolb.2018.00065

**Published:** 2018-07-13

**Authors:** Jayangika N. Dahanayake, Katie R. Mitchell-Koch

**Affiliations:** Department of Chemistry, Wichita State University, Wichita, KS, United States

**Keywords:** viscosity, protein dynamics, hydration dynamics, solvation shell, CALB, Markov state model, Kramers' theory, non-aqueous enzymes

## Abstract

Solvation is critical for protein structural dynamics. Spectroscopic studies have indicated relationships between protein and solvent dynamics, and rates of gas binding to heme proteins in aqueous solution were previously observed to depend inversely on solution viscosity. In this work, the solvent-compatible enzyme *Candida antarctica* lipase B, which functions in aqueous and organic solvents, was modeled using molecular dynamics simulations. Data was obtained for the enzyme in acetonitrile, cyclohexane, *n-*butanol, and *tert-*butanol, in addition to water. Protein dynamics and solvation shell dynamics are characterized regionally: for each α-helix, β-sheet, and loop or connector region. Correlations are seen between solvent mobility and protein flexibility. So, does local viscosity explain the relationship between protein structural dynamics and solvation layer dynamics? Halle and Davidovic presented a cogent analysis of data describing the global hydrodynamics of a protein (tumbling in solution) that fits a model in which the protein's interfacial viscosity is higher than that of bulk water's, due to retarded water dynamics in the hydration layer (measured in NMR τ_2_ reorientation times). Numerous experiments have shown coupling between protein and solvation layer dynamics in site-specific measurements. Our data provides spatially-resolved characterization of solvent shell dynamics, showing correlations between regional solvation layer dynamics and protein dynamics in both aqueous and organic solvents. Correlations between protein flexibility and inverse solvent viscosity (1/η) are considered across several protein regions and for a rather disparate collection of solvents. It is seen that the correlation is consistently higher when local solvent shell dynamics are considered, rather than bulk viscosity. Protein flexibility is seen to correlate best with either the local interfacial viscosity or the ratio of the mobility of an organic solvent in a regional solvation layer relative to hydration dynamics around the same region. Results provide insight into the function of aqueous proteins, while also suggesting a framework for interpreting and predicting enzyme structural dynamics in non-aqueous solvents, based on the mobility of solvents within the solvation layer. We suggest that Kramers' theory may be used in future work to model protein conformational transitions in different solvents by incorporating local viscosity effects.

## Introduction

**Figure 1:**
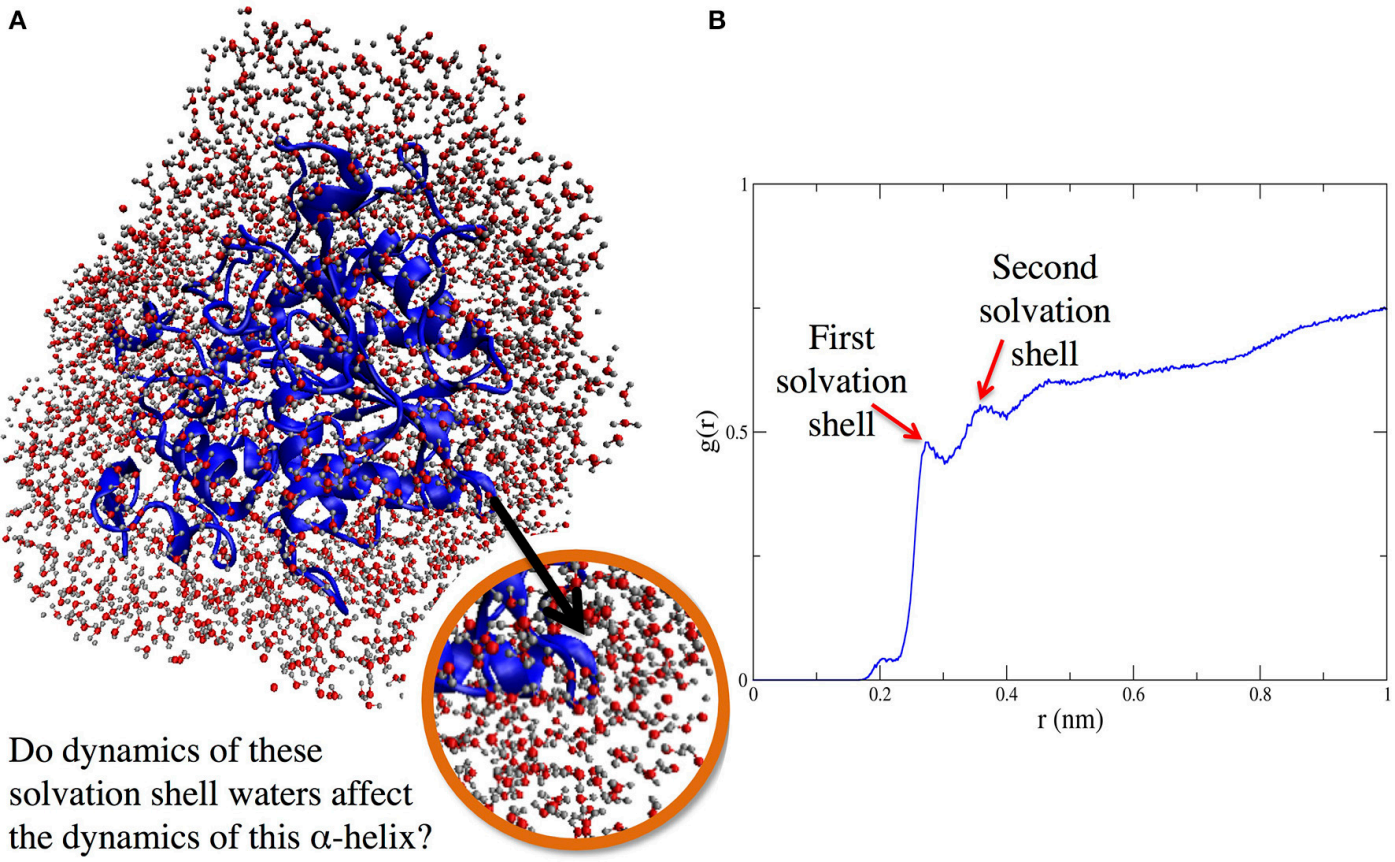
**(A)** (left side) Protein solvation shell (water around CALB). **(B)** (right side) Radial distribution function, g(r), showing solvent layering around α5 helix as a function of distance (in nm) from nearest protein atom of helix α5.

When it comes to protein structure-dynamics, it is clear that solvent matters. In fact, decades of experiments have shown that solvent *dynamics* and protein dynamics are intimately related. Here, we hypothesize that what really matters in the protein-solvent dynamics relationship can be summarized by that old real estate maxim: *location, location, location*. Our work evaluating methodologies for simulation of enzymes in organic solvent revealed that the location of crystallographic waters (i.e., *which* crystallographic waters) kept during simulations influences the conformational dynamics of the enzyme CALB (Dahanayake et al., 2016). The present work focuses on the local connection between solvent dynamics and protein dynamics, looking specifically at whether and how a protein region's flexibility or stability may depend on the dynamics of solvent in the solvation layer surrounding different portions of the proteins: for example, solvent in the immediate surroundings of an alpha helix (Figure 1).

Protein-solvent dynamics connections were observed in solvent-dependent gas-binding rates to heme, myoglobin, and hemoglobin. Beece et al. used flash photolysis to measure gas binding rates to heme proteins in aqueous solutions of different viscosities (Beece et al., 1980). Their data showed that transition rates between different protein conformations are a function of solvent viscosity. Since ligand diffusion and protein dynamics are examples of thermal, or Brownian, motion, the dependence of protein dynamics on solvent dynamics can be understood to arise from the friction of the local solvation environment, represented as solvent viscosity (albeit a local or interfacial measure of viscosity).

In myoglobin, Kramers' theory fits the kinetics describing the process of the gas molecule leaving the solvent and moving in to a “cage” near the heme iron (Beece et al., 1980). In Kramers' theory, Brownian motion leads to escape over a potential barrier (akin to an activation energy), where the rate is described by

(1) $$\begin{array}{r} k\;\alpha \;1/\eta \;e^{-H*/RT}, \end{array}$$

where η is the solvent viscosity and H^*^ is the potential energy barrier (R is Boltzmann/gas constant and T is temperature). Note that recently, Kramers' theory in the high friction limit has been used to describe the rates of protein conformational transitions, which are inversely proportional to friction, ζ, often represented as viscosity, η (Frauenfelder and Wolynes, 1985; Schlitter, 1988; Plotkin and Onuchic, 2002; Hagen, 2010; Frank and Andricioaei, 2016). Protein dynamics, therefore, should be facilitated by locally lower friction environments at the protein-solvent interface. Protein unfolding, on the other hand, should be inhibited by high friction environments represented by a slow-moving (viscous) solvation shell. In the Stokes-Einstein limit, the friction is reduced to a viscosity term, η, which is related to local solvent reorientation times (τ_2_, often measured by NMR spectroscopy) by τ_2_(surface)/τ_2_(bulk) = η(surface)/η(bulk) (Halle and Davidovic, 2003) or diffusion coefficients (D) by D(bulk)/D(surface) = η(surface)/η(bulk). A word of caution is required: Stokes-Einstein-Debye diffusion is not valid in all systems (Beddard et al., 1981; Turton and Wynne, 2014). Turton and Wynne investigated diffusion of guanidine hydrochloride in polar solvation (under different concentrations in water) and CS_2_ in non-polar solvation (hexadecane). In each case, Stokes-Einstein-Debye rotational diffusion failed, with authors suggesting that “diffusion of molecular-size particles is dominated by local interactions that decouple the diffusivity from bulk viscosity,” with effects arising primarily from interactions with the first solvation shell (Turton and Wynne, 2014).

Regardless of whether a protein's diffusive motion is taking place in the Stokes-Einstein-Debye limit, solvent dynamics at the protein surface are descriptors of the friction surrounding the moving particle, which perhaps can be deemed effectively the “local viscosity.” The protein's thermal motion takes place within a constrained, quasi-harmonic potential, which gives rise to its observed structural and conformational dynamics. The thermal, or Brownian, motion of the protein region depends on the local friction: thus, it is influenced by its local solvent cage, or solvation layer. This present work focuses on local descriptors of solvation layer mobility that can be experimentally measured, examining how they are correlated with enzyme structural dynamics.

The observations and analysis by Beece et al. in their seminal paper titled “Solvent Viscosity and Protein Dynamics” (Beece et al., 1980) led to the formulation of solvent-slaving theory (Fenimore et al., 2002). Solvent-slaving theory postulates that protein processes fall into two categories: solvent-slaved and non-slaved classes (Fenimore et al., 2002). The rate coefficient for a solvent-slaved process, *k(T)* is given by

(2) $$\begin{array}{r} k\left(T\right)=k_{diel}\left(T\right)/n\left(T\right) \end{array}$$

where *k*_*diel*_*(T)* is the dielectric relaxation rate of the solvent, which describes the average reorientational dynamics of the bulk liquid. Meanwhile, *n(T)* is a process-dependent proportionality constant that putatively takes into effect the hydration shell, and in the case of myoglobin, is stated by Fenimore et al. to be related to the number of conformational substates that lead to channel opening for CO passage in and out of the protein (Fenimore et al., 2002).

In spectroscopic studies, Marx and co-workers showed possible support for this relationship between protein dynamics and the dynamics of *bulk water* (Baer et al., 2006), while also showing in studies that the *interfacial* dynamics of water (protein-water hydrogen bond dynamics) may mediate a peptide's temperature dependent behavior (Rousseau et al., 2004; Schreiner et al., 2004).

Although correlations between protein dynamics and bulk solvent viscosity have been found in aqueous solutions (Beece et al., 1980), there is also evidence that the properties of the solvent shell matter. For example, the global hydrodynamics of proteins, that is, the tumbling of globular proteins in water, was explained by Halle and Davidovic to depend on hydration layer dynamics (Halle and Davidovic, 2003). Prior to their work, it was observed that the tumbling rate of proteins in water could be interpreted to give proteins an apparent volume higher than their known volume (by crystallographic structures). In other words, proteins (approximated to be spherical) rotate and diffuse more slowly than would be predicted from their size and the viscosity of bulk water, according to Stokes-Einstein relationships for translation (D_T_) and rotation (D_R_):

(3) $$\begin{array}{r} D_{T}=\;\frac{k_{B}T}{6\pi \eta r}\;and\;D_{R}=\;\frac{k_{B}T}{8\pi \eta r^{3}} \end{array}$$

where η is the solvent shear viscosity and *r* is the radius. To explain this result, it had been postulated that proteins tumble with an intact shell of bound water, supporting an iceberg model of solvation that gives proteins a larger apparent volume. Halle and Davidovic recognized that the rigid hydration shell model is in conflict with measured values of hydration shell residence times, which are on the order of 20–40 ps, much faster than the nanosecond-scale global motions of proteins. Rather, they recognized that the waters in the hydration layer, due to their retarded dynamics relative to bulk water, provide a higher friction environment (dubbed interfacial viscosity) through which the protein moves. Halle and Davidovic used water reorientation times (τ_2_, measured through ^17^O magnetic dispersion relaxation measurements) as indicators of the local, interfacial viscosity (η), where

(4) $$\begin{array}{r} \frac{\tau_{2,\;interface}}{\tau_{bulk}}=\;\frac{\eta_{interface}}{\eta_{bulk}}. \end{array}$$

This relationship provides a value of the interfacial viscosity:

(5) $$\begin{array}{r} \eta_{interface}=\;\eta_{bulk}\;\frac{\tau_{2,\;interface}}{\tau_{bulk}} \end{array}$$

describing the solvation environment in which the protein undergoes diffusive motion (Halle and Davidovic, 2003). This interpretation has provided a convincing model to explain protein (global) hydrodynamics.

Although the hydration dynamics around globular proteins were measured to undergo a relatively generic retardation (Mattea et al., 2008), it has been shown more recently through site-specific probes and spatially-resolved techniques that the dynamics of waters in the hydration layer are heterogeneous. Some regions of the protein are solvated by waters that are rather dramatically slowed relative to bulk dynamics, whereas other waters undergo very modest retardation at the protein interface. As might be expected, waters in the concave interiors of protein (bound and active site waters) are quite slow (Hua et al., 2006; Sterpone et al., 2012). However, exterior surface waters have also been shown to exhibit a range of dynamics within the protein hydration layer (Nucci et al., 2011; King and Kubarych, 2012; Qin et al., 2016). Furthermore, spectroscopic measurements have shown that local protein dynamical fluctuations are coupled to the solvation layer's dynamical fluctuations (King et al., 2012; Qin et al., 2016).

Beyond effects on dynamics, hydration dynamics appear to serve other roles in protein function, such as molecular recognition (Caro et al., 2017). In work on catalytic nanoparticles, Han et al. showed that catalytically-active nanoparticles had spectroscopic signatures of hydration dynamics that are similar to (catalytically-active) enzymes, raising interesting questions about the role of hydration shell dynamics in catalytic and enzymatic function (Stals et al., 2016). The regional differences in hydration dynamics around the protein surface have been connected to molecular recognition events within the protein (Pal and Zewail, 2004; Young et al., 2007). For example, intriguing work by Havenith and co-workers, using terahertz spectroscopy and molecular dynamics (MD) simulations, revealed a hydration funnel with slowing hydrogen bond dynamics at the protein surface as substrate approaches the active site (Nibali and Havenith, 2014). The tie between local solvent dynamics and regional protein dynamics (flexibility/stability) may explain another function of the heterogeneous hydration dynamics that have been experimentally observed around proteins. Our hypothesis is that the dynamics of the local hydration shell around regions of a protein (for instance, an α-helix) modulate the dynamics of that region. This is achieved by altering the solvation layer friction, or effective local viscosity, through which the protein region moves. Diffusive motion is dependent on the solvent environment. Rates between protein conformational states, then, are dependent on a measure of the local friction/viscosity and can be described using Kramers' theory (Equation 1).

A definitive description of the relationship between protein dynamics and solvent dynamics is an area of active investigation for the scientific community. The problem is quite complex, although experiments clearly demonstrate that protein and solvent dynamics are connected. The hypothesis and theory discussion presented here refines and combines several seminal models describing the protein-solvent dynamics relationship, with an eye toward describing not only protein dynamics in aqueous solutions, but in organic solvents, too. We present new results for protein and solvation shell dynamics for the protein *Candida antarctica* lipase B (CALB) in water, acetonitrile, *n-*butanol, *tert-*butanol, and cyclohexane, and evaluate connections between *regional* protein and solvent dynamics in the data.

**Figure 2:**
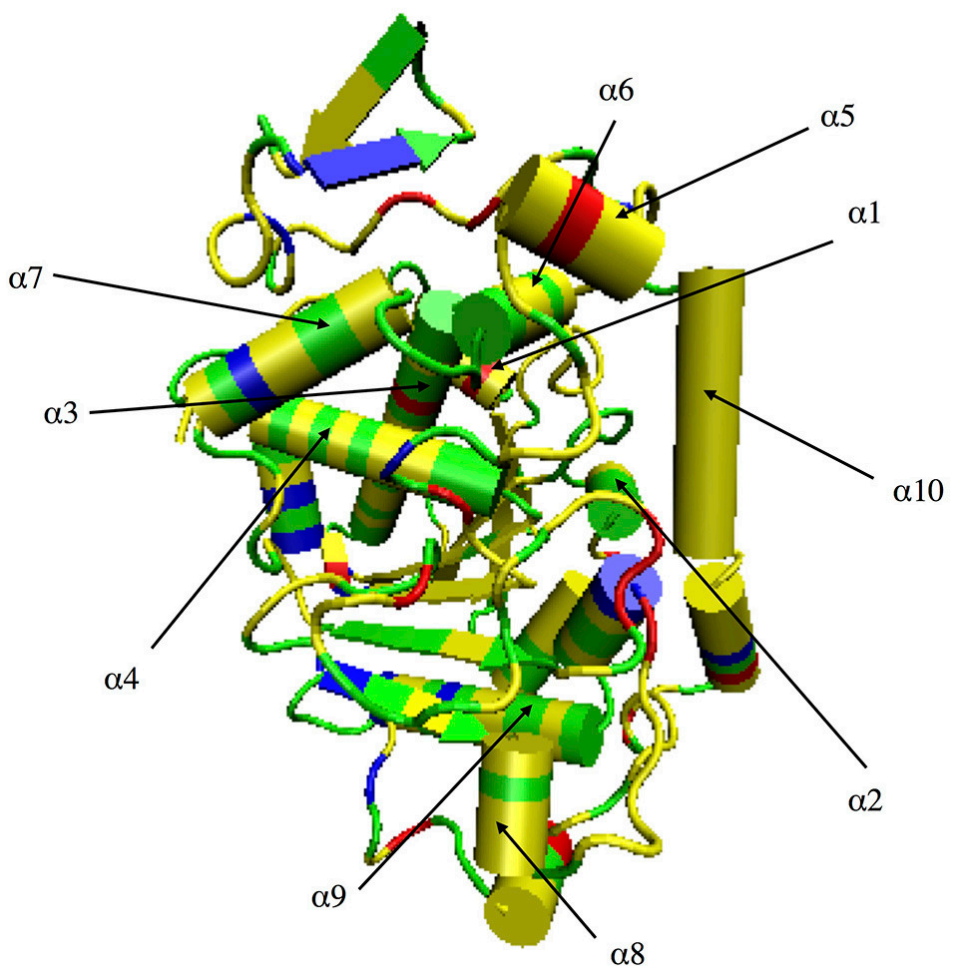
CALB surface color-coded by amino acid type. Yellow indicates hydrophobic residues, green indicates polar residues, red indicates acidic residues, and blue corresponds to basic residues.

CALB, shown in Figure 2, is an example of a solvent-compatible enzyme, or a protein that retains its folded structure and catalytic ability in solvents other than water. Only a fraction of proteins are solvent-compatible. The lipase studied here is fairly unique, in that it can function under strictly non-aqueous conditions, rather than being limited to organic-aqueous mixtures, as a number of solvent-compatible proteins are. Because of its solvent compatibility, the enzyme CALB was used as a model in this study, in order to evaluate a possible function for heterogeneous solvation shell dynamics: to modulate regional protein dynamics. CALB is a 33.273 KDa enzyme (Uppenberg et al., 1994) that behaves as an esterase in solution with small molecule reactants, but can undergo interfacial activation (classic lipase behavior) with more bulky substrates (Zisis et al., 2015). CALB is a versatile biocatalyst that has been used in water and organic solvents for various reactions, including capsaicin hydrolysis, phenolic acid esterification, octyl-β-glucoside synthesis, and alkyl ester production (Ljunger et al., 1994; Guyot et al., 1997; Anderson et al., 1998; Duarte et al., 2000; Deng et al., 2005; Yu and Lutz, 2010).

The use of enzymes in organic solvent increases solubility of hydrocarbons (relative to traditional aqueous biocatalysis), and can also tune the regio-, chemo-, and stereoselectivity of reactions (Kitaguchi et al., 1989; Wescott and Klibanov, 1994; Gotor, 1999; Klibanov, 2001), while suppressing competing hydrolysis reactions (Bistline et al., 1991). However, a drawback of non-aqueous enzyme catalysis is decreased flexibility of the protein, which leads to reduced activity (Clark, 2004). It has been observed that addition of small amounts of water increases both the kinetics and flexibility of proteins in organic solvent. Molecular dynamics studies by Wedberg et al. of CALB in methanol, *tert-*butyl alcohol, methyl *tert-*butyl ether, and hexane solvents with various fractions of water showed that residence times of solvent decreased as water fraction (more specifically, water activity) increases, concomitant with higher enzyme flexibility (Wedberg et al., 2012). Other simulations studies of CALB in organic solvent tied reduced enzyme flexibility to the formation of water networks with slow dynamics (Trodler and Pleiss, 2008). Experimental work shows that the addition of salt during enzyme lyophilization (“salt activation”) leads to markedly higher enzyme activity in organic solvents, accompanied by increased mobility of residual waters in the solvation layer (Eppler et al., 2006). The work presented herein examines solvation layer dynamics, presenting the first spatially-resolved mapping of organic solvent dynamics around the enzyme. Solvent and protein dynamics are also examined in CALB's native solvent, water, for a better understanding of how solvation layer mobility may affect protein dynamics and function.

## Methodology

### Simulation details

Molecular dynamics simulations of CALB enzyme in explicit (atomistic model) solvent were carried out. Four different organic solvents: acetonitrile, *n-*butanol, *tert-*butanol, and cyclohexane, comprising a range of solvent viscosities (given in Table 1), were selected as the non-aqueous media in this study. Further, CALB enzyme was studied in the presence of water media for comparison. The starting coordinates of CALB enzyme were taken from the X-ray crystallographic structure (PDB ID: 1TCA). Six buried crystallographic water molecules identified by Uppenberg et al. (1994) were kept, as it was concluded in our previous studies (Dahanayake et al., 2016) that this leads to fastest equilibration in non-aqueous simulations. All the simulations were performed using GROMACS (version 4.6.3) (Kamatari et al., 1996) software package.

**Table 1:** Diffusion coefficients, experimental viscosity values (Haynes et al., 2015), and apparent viscosity for each solvent model (based on Equation 3).

	**Experimental viscosity (mPa.s)**	**Experimental diffusion coefficient (× 10^−5^ cm^2^/s)**	**Model diffusion coefficient (× 10^−5^ cm^2^/s)**	**Percent error for model diffusion coefficient (%)**	**Model apparent viscosity (mPa.s)**
Water	0.89	2.3	2.49	8.3	0.79
*n-*butanol	2.95	0.46	0.46	14.4	2.92
*t-*butanol	3.35	0.30	0.29	1.1	3.44
Acetonitrile	0.39	4.04	4.62	2.6	0.34
Cyclohexane	0.98	1.47	1.05	28.8	1.38

OPLS-AA force field (Jorgensen et al., 1996) was used for CALB enzyme and for all the organic solvents used. For validation that results are not force field dependent, simulations were also run using Amber03 forcefield for protein and solvent. Solvent models that reproduce experimental solvent dynamics well were intentionally selected (with OPLS-AA providing best agreement among commonly-used protein force field suites), as the solvent dynamics are a key focus of this paper. Table 1 provides a comparison of diffusion coefficients from experiments and solvent models at 298 K. As can be seen, cyclohexane's diffusion and viscosity do deviate from experimental values. Our modeling of correlations between protein dynamics and solvent dynamics mitigates this by using the model's apparent viscosity (also listed in Table 1). SPC/E water model (Berendsen et al., 1987) was used to represent water. This model's values of diffusion and reorientational dynamics are similar to experiment, and SPC/E water was recently shown to reproduce experimentally measured protein-water hydrogen bond lifetimes (King et al., 2014). The enzyme was centered in a cubic periodic box with a minimum distance of 1.0 nm between protein and any side of the box. Next, for the simulations with organic solvents, the enzyme was solvated in organic solvent, and one sodium counter ion was added to CALB by replacing one solvent molecule, in order to obtain a neutral charge. For the simulations with water, the enzyme was solvated in water, and Na^+^ and Cl^−^ ions were added, replacing water molecules, to neutralize the systems at a 0.15 M salt concentration. Bond lengths were constrained using LINCS algorithm (Hess et al., 1997). Electrostatic interactions were calculated using Particle Mesh Ewald summation (Darden et al., 1993). For long-range interactions, a grid spacing of 0.12 nm combined with an interpolation order of 4 was used. Van der Waals interactions were calculated using a 1.4 nm cut-off. Energy minimization was done using steepest descent algorithm (Arfken, 1985) to remove clashes between atoms that were too close. Position restraints were used on heavy atoms during annealing, when the system was gradually heated from 50 to 300 K in 200 ps. Systems were equilibrated in the NPT ensemble for 20 ns at 300 K using V-rescale thermostat (Bussi et al., 2007) and at 1 bar using Berendsen barostat (Berendsen et al., 1984) for conditions similar to *in vitro* catalysis (Anderson et al., 1998). Finally, the production runs were done in NVT ensembles at 300 K using Nosé-Hoover thermostat for a canonical ensemble (Nose, 1984; Hoover, 1985). Note that *tert-*butanol simulations were run at 301 K in order to have the system above the solvent's melting point. Results from molecular dynamics simulations were obtained after production runs comprising six trajectories per solvent, each generated using different randomly assigned initial velocities. Among them, five trajectories were obtained with 100 ns simulation times, and the sixth trajectory was obtained with a 500 ns simulation time for a total of 1 μs. Long trajectories were carried out with XSEDE computing resources (Towns et al., 2014). Although solvent dynamics take place on picosecond timescales and can be adequately sampled with shorter trajectories, a longer timescale such as this is valuable when analyzing protein conformational dynamics (Verma and Mitchell-Koch, 2017).

### Analysis

MD simulations were analyzed to characterize protein dynamics and solvent dynamics at the CALB enzyme interface. In order to characterize solvent dynamics at the CALB enzyme interface, regional protein-solvent hydrogen bond lifetimes (HBLTs), solvation layer residence times, and diffusion times of solvation shell (first solvation shell) solvent molecules were calculated. Regional solvent dynamics were found around each separate secondary structure; these were calculated for solvents within the local solvation layer (within a fixed distance from atoms in each α-helix, β-sheet, and connector region). For statistical sampling, analyses (detailed below) were block-averaged over the multiple trajectories. For the five trajectories with 100 ns simulation times, these blocks were considered for the last 50 ns, using 25 ns time blocks. For the sixth trajectory with 500 ns simulation time, these blocks were considered for 225–250 ns and 475–500 ns time blocks (giving 12 blocks, 300 ns total for analysis of solvent dynamics). Uncertainties are reported for the solvent dynamics parameters at the 95% confidence level, using the student *t-*test (Shoemaker et al., 1996).

In order to define the first solvent shell for each solvent, the radial distribution functions of solvent around side chain atoms were calculated around each α-helix. Figures S1A–D show the radial distribution functions of each organic solvent studied around side chain atoms of several exterior alpha helices (i.e., α1, α5, α8, and α10). For acetonitrile, the radial distribution functions were calculated for the acetonitrile nitrogen atom around CALB side chain atoms. For alcohols, the radial distribution functions were calculated for the alcohol oxygen atom around CALB side chain atoms (note that OPLS-AA is an all-atom force field for both protein and solvent). It was found that for all regions but helices α4, α6, and α8, the first solvation shell is contained within 5 Å for acetonitrile, 6 Å for *n-*butanol, 4 Å for *tert-*butanol, and 7 Å for cyclohexane. For α4, α6, and α8, the first solvation shell is contained within 7 Å for acetonitrile, 8 Å for *n-*butanol, 6 Å for *tert-*butanol, and 9 Å for cyclohexane.

GROMACS software was used to obtain hydrogen bond auto-correlation functions (van der Spoel et al., 2006), which were analyzed graphically to obtain hydrogen bond lifetimes (Luzar and Chandler, 1996). Hydrogen bond correlation times are presented as the *1/e* times in Tables 2–6 (as suggested by King et al., 2014).

**Table 2:** Solvent dynamics of water around each α-helix.

	**α1 (Ext)**	**α2 (Int-Ext)**	**α3 (Int-Ext)**	**α4 (Int)**	**α5 (Ext)**	**α6 (Int)**	**α7 (Int-Ext)**	**α8 (Ext)**	**α9 (Int-Ext)**	**α10 (Ext)**
Hydrogen bond lifetime (ps)	22.6 ± 0.4	54.5 ± 5.3	63.2 ± 10.3	478 ± 96	25.3 ± 3.9	459 ± 110	20.6 ± 2.0	25.1 ± 0.5	36.8 ± 2.9	43.3 ± 2.3
Diffusion coefficient (10^−5^ cm^2^/s)	1.668 ± 0.029	1.116 ± 0.016	0.986 ± 0.021	0.172 ± 0.006	1.384 ± 0.044	0.586 ± 0.054	1.258 ± 0.097	1.228 ± 0.034	1.059 ± 0.022	1.454 ± 0.009
Residence time (ps)	45.7 ± 0.7	56.2 ± 1.4	52.6 ± 1.2	75.8 ± 4.3	42.5 ± 0.6	63.2 ± 1.6	46.1 ± 1.6	41.9 ± 0.8	59.5 ± 1.2	44.3 ± 0.3

*Interior (Int) and exterior (Ext) status of the protein regions are indicated. (Calculated diffusion coefficient for the bulk solvent = 2.49 × 10^−5^ cm^2^/s)*.

**Table 3:** Solvent dynamics of *n-*butanol around each α-helix.

	**α1 (Ext)**	**α2 (Int-Ext)**	**α3 (Int-Ext)**	**α4 (Int)**	**α5 (Ext)**	**α6 (Int)**	**α7 (Int-Ext)**	**α8 (Ext)**	**α9 (Int-Ext)**	**α10 (Ext)**
Hydrogen bond lifetime (ps)	721 ± 17	1,202 ± 241	792 ± 78	1652 ± 218	422 ± 34	–	884 ± 42	334 ± 13	1063 ± 113	746 ± 51
Diffusion coefficient (10^−5^ cm^2^/s)	0.417 ± 0.006	0.271 ± 0.004	0.279 ± 0.003	0.167 ± 0.002	0.368 ± 0.004	0.266 ± 0.005	0.338 ± 0.004	0.369 ± 0.004	0.254 ± 0.011	0.446 ± 0.002
Residence time (ps)	29.3 ± 1.1	23.3 ± 1.7	25.1 ± 1.0	26.3 ± 1.1	35.8 ± 1.1	31.1 ± 1.2	28.7 ± 1.4	38.7 ± 0.6	20.0 ± 0.3	34.3 ± 0.7

*(Calculated diffusion coefficient for the bulk solvent = 0.461 × 10^−5^ cm^2^/s)*.

**Table 4:** Solvent dynamics of *tert-*butanol around each α-helix.

	**α1 (Ext)**	**α2 (Int-Ext)**	**α3 (Int-Ext)**	**α4 (Int)**	**α5 (Ext)**	**α6 (Int)**	**α7 (Int-Ext)**	**α8 (Ext)**	**α9 (Int-Ext)**	**α10 (Ext)**
Hydrogen bond lifetime (ps)	748 ± 29	660 ± 49	705 ± 32	–	270 ± 65	–	177 ± 36	1475 ± 218	734 ± 39	1342 ± 182
Diffusion coefficient (10^−5^ cm^2^/s)	0.223 ± 0.005	0.192 ± 0.007	0.151 ± 0.007	0.099 ± 0.010	0.180 ± 0.003	0.136 ± 0.005	0.136 ± 0.006	0.186 ± 0.006	0.123 ± 0.004	0.194 ± 0.005
Residence time (ps)	22.3 ± 1.5	25.0 ± 3.6	29.8 ± 3.2	45.4 ± 1.8	27.2 ± 2.5	34.6 ± 1.6	33.6 ± 4.8	26.8 ± 1.6	36.3 ± 4.1	23.4 ± 0.7

*(Calculated diffusion coefficient for the bulk solvent = 0.292 × 10^−5^ cm^2^/s)*.

**Table 5:** Solvent dynamics of acetonitrile around each α-helix.

	**α1 (Ext)**	**α2 (Int-Ext)**	**α3 (Int-Ext)**	**α4 (Int)**	**α5 (Ext)**	**α6 (Int)**	**α7 (Int-Ext)**	**α8 (Ext)**	**α9 (Int-Ext)**	**α10 (Ext)**
Hydrogen bond lifetime (ps)	37.7 ± 0.8	19.5 ± 0.3	73.6 ± 1.3	–	210 ± 47	418 ± 107	36.8 ± 0.6	–	216 ± 19	63.6 ± 0.4
Diffusion coefficient (10^−5^ cm^2^/s)	3.23 ± 0.05	2.32 ± 0.07	1.88 ± 0.06	1.68 ± 0.07	3.01 ± 0.05	1.47 ± 0.08	2.79 ± 0.06	3.04 ± 0.07	1.47 ± 0.06	3.08 ± 0.03
Residence time (ps)	20.3 ± 0.5	33.6 ± 0.6	32.0 ± 0.9	37.2 ± 1.0	29.8 ± 0.6	39.2 ± 1.0	32.5 ± 1.0	29.6 ± 0.4	37.5 ± 0.4	25.0 ± 0.2

*(Calculated diffusion coefficient for the bulk solvent = 4.62 × 10^−5^ cm^2^/s)*.

**Table 6:** Solvent dynamics of cyclohexane around each α-helix.

	**α1 (Ext)**	**α2 (Int-Ext)**	**α3 (Int-Ext)**	**α4 (Int)**	**α5 (Ext)**	**α6 (Int)**	**α7 (Int-Ext)**	**α8 (Ext)**	**α9 (Int-Ext)**	**α10 (Ext)**
Diffusion coefficient (10^−5^ cm^2^/s)	0.76 ± 0.02	0.67 ± 0.02	0.67 ± 0.02	0.64 ± 0.02	0.95 ± 0.02	0.65 ± 0.02	0.74 ± 0.01	0.78 ± 0.02	0.64 ± 0.01	1.04 ± 0.01
Residence time (ps)	24.1 ± 1.0	29.4 ± 0.9	28.5 ± 0.9	38.6 ± 0.9	20.8 ± 1.0	39.2 ± 1.2	24.1 ± 0.6	23.8 ± 0.3	32.9 ± 0.5	15.8 ± 0.4

*(Calculated diffusion coefficient for the bulk solvent = 1.05 × 10^−5^ cm^2^/s)*.

A Fortran code was developed to calculate residence times of solvation shell solvent molecules. The residence time describes how long a solvent molecule stays in the protein solvation layer before leaving. A survival probability time correlation function, *C*_*res*_*(t)*, was calculated, in which a solvent molecule residing in the layer is assigned a value of 1 at time *t* (*h(t)* = 1), and a value of 0 when it leaves the hydration layer (*h(t)* = 0), giving:

(6) $$\begin{array}{r} C_{res}\left(t\right)=\langle h\left(t\right)\cdot h\left(0\right)\rangle \end{array}$$

where the brackets denote averaging over all the solvent molecules in the solvation layer across multiple time blocks. The residence time is fit to the time when *C*_*res*_*(t)* = 1/e and residence times were also calculated by histogramming the time needed by a solvent molecule to leave the protein solvation layer. Histogram and correlation time values were found to be statistically equivalent, and values presented in Tables 2–6 are from the histogram averages.

GROMACS software was used to calculate self-diffusion coefficients of solvent molecules in the solvation layer from mean-square displacements (MSD) using the Einstein relationship,

(7) $$\langle r^{2}\rangle =\begin{array}{c} \lim\\ t\to \infty \end{array}2\alpha Dt$$

where < *r*^2^> is the mean square displacement, α is the dimensionality in the diffusion process, and *D* is the diffusion coefficient.

The (bulk solvent) viscosity of the model was calculated using Stokes-Einstein relationships (Equation 3) to be:

(8) $$\begin{array}{r} \eta_{model}\approx \;\eta_{experiment}\;\frac{D_{experiment}}{D_{model}}, \end{array}$$

where the values of viscosity (η) and diffusion (D) are taken from experimental data (Table 1) and model diffusion coefficients come from simulations of bulk solvent with the OPLS-AA force field. Local viscosities of the solvation layer (η_*local*_) are calculated from diffusion constants around each protein region, as

(9) $$\begin{array}{r} \eta_{local}\approx \;\eta_{bulk}\;\frac{D_{bulk}}{D_{local}}, \end{array}$$

where *D*_*bulk*_ is the diffusion coefficient of the model as bulk solvent and *D*_*local*_ is the diffusion coefficient of the solvent within the solvation layer surrounding a given region of the protein.

In order to comprehensively analyse protein dynamics, the root mean square fluctuation (RMSF) of atomic positions in the trajectory was calculated and RMSF peak integration was done to obtain a statistical sampling for the protein dynamics. Further, a hidden Markov state model was used to calculate transition rates between conformations of CALB in each solvent.

**Figure 3:**
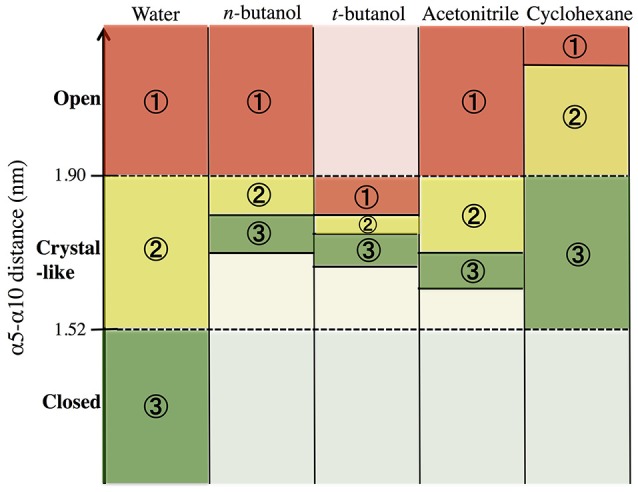
Distribution of states obtained using hidden Markov state model in the conformational sampling of CALB enzyme.

In order to build and analyze a Markov state model, PyEMMA software (PyEMMA 2.4 version) (Scherer et al., 2015) was used. Metastable (long-lived) states, also referred to here as conformations, were obtained using the Perron Cluster Cluster Analysis (PCCA) algorithm. Aqueous CALB is known to occupy three conformations: open, crystallographic, and closed; defined by the α5–α10 nearest distance (Zisis et al., 2015). For this reason, we specified that the PCCA algorithm analyze the trajectory for transitions among three states, but the definition of states was achieved through hidden Markov analysis. We compared the three aqueous states found in hidden Markov analysis to the three experimentally-defined states, and found them to be in agreement. For conformational sampling in organic solvent, a three state model was also specified, so that analysis could be compared with aqueous results. Figure 3 shows the α5–α10 distances that define the three conformational states in each solvent. As can be seen, conformations are solvent-dependent. Lag times, τ, for Markov analysis were determined by varying the length until the implied timescales no longer change with τ (see Figure S2). Since conformational sampling is solvent-dependent, the lag time values (τ) were evaluated independently for all solvents. However, a value of τ = 15 ns was found to be suitable for all solvents. Conformational transition rates were obtained by coarse-graining a kinetic model between these three conformational states using hidden Markov state model. Error bars for conformational transition rates in each solvent were determined by bootstrapping using five data sets, reported as standard deviation.

## Results

Protein trajectories were analyzed for stability, and all proteins remain folded and similar to the crystallographic structure (PDB ID: 1TCA) throughout the length of all simulations. Stability of the protein in each solvent was confirmed by evaluating RMSD over time (relative to the crystal structure), which indicates an equilibrated folded structure in all circumstances (Figures S3, S4). The hydrophilic and hydrophobic surface areas were also evaluated to compare protein structures across solvents (Figure S5). As observed by Li et al., the hydrophilic surface area reduces somewhat in organic solvent, relative to the crystal and aqueous structures (Li et al., 2010). Radius of gyration values also indicate similar, stable, folded structures across all conditions (Table S1).

**Figure 4:**
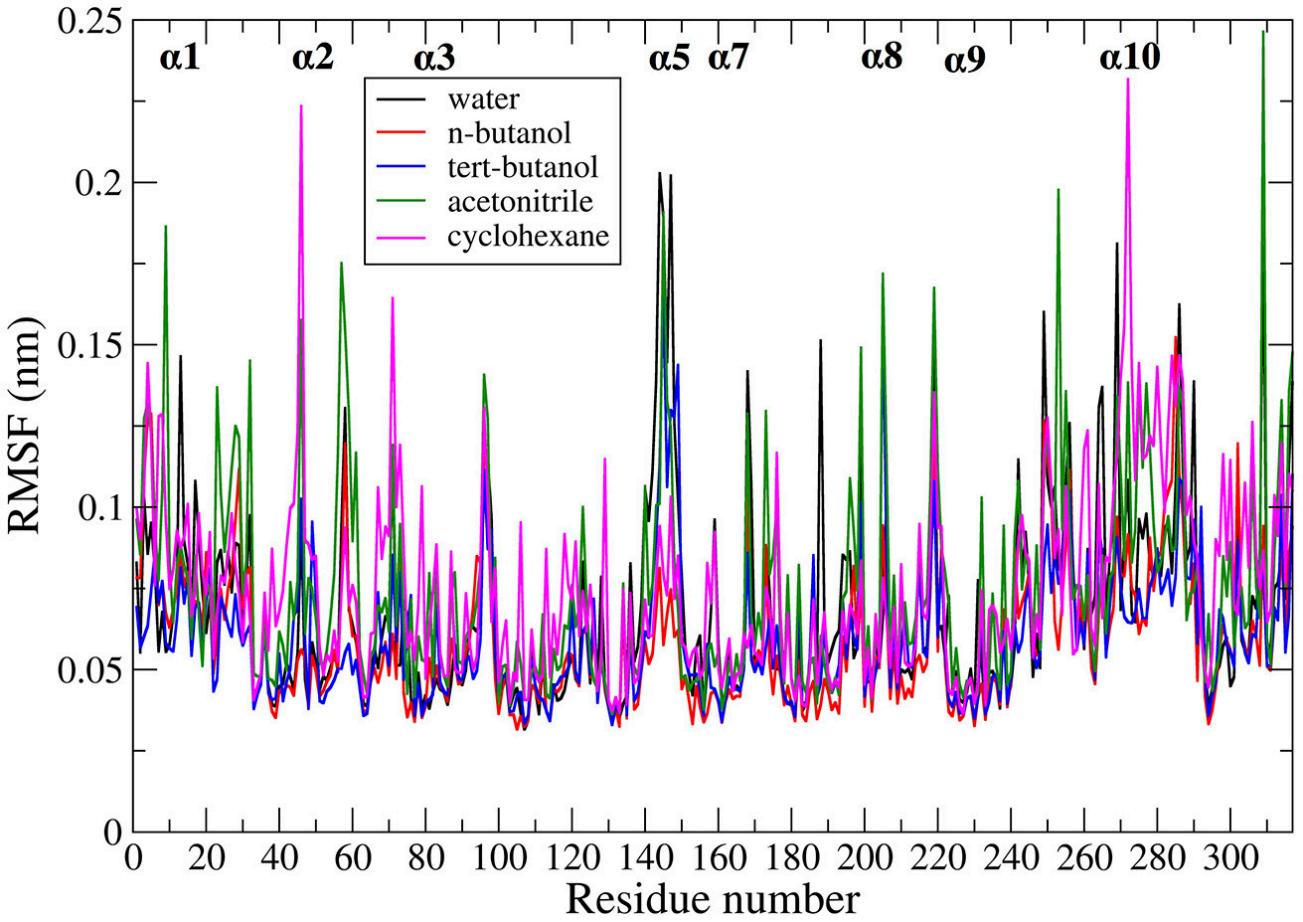
Comparison of RMSF averaged over all the ensembles, in the presence of different solvents studied: water (black), *n-*butanol (red), *tert-*butanol (blue), acetonitrile (green), and cyclohexane (purple).

Protein dynamics by residue were examined by calculating root mean square fluctuation (RMSF). The RMSF per residue values are presented in Figure 4 for the five solvents studied (water, *n-*butanol, *tert-*butanol, acetonitrile, and cyclohexane). The RMSF values for simulations run in Amber force field are in Figure S6 of Supporting Information, and it can be seen that the protein dynamics are virtually identical in every solvent, regardless of force field. High RMSF values in Figures 4 and S6 indicate high flexibility, and it can be seen that regions of protein flexibility depend on the solvent. Of special interest is the α5 helix region, residues 142–146 and connectors (comprising residues 140–150), which is the primary region involved in conformational transitions that allow for substrate binding and release. The α5 region is shown to be most flexible in water and acetonitrile, while motion is significantly suppressed in *n-*butanol and cyclohexane. It is interesting to note that not all protein motions are damped in organic solvent. For instance, the α2 and α10 regions display their highest flexibility in cyclohexane. The flexibility of CALB in acetonitrile is generally high, and fairly similar to water. In contrast, the two butanol solvents overall display the most hindered protein dynamics. These are the two most viscous solvents in the study.

**Figure 5:**
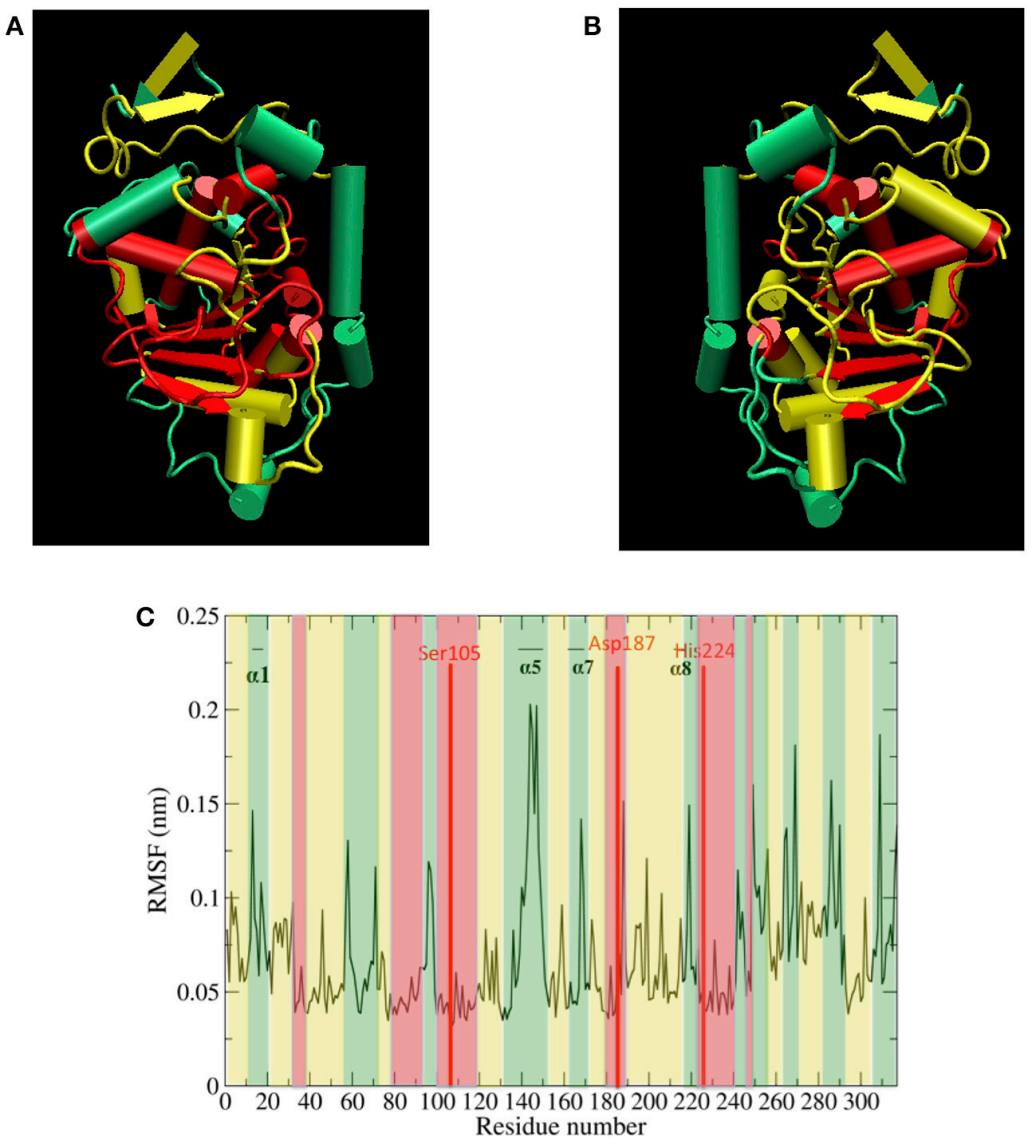
**(A)** (top left) Protein hydration shell water diffusion coefficients mapped to CALB structure (Green > 1.5 × 10^−5^ cm^2^/s, yellow 1.0–1.5 × 10^−5^ cm^2^/s, red < 1.0 × 10^−5^ cm^2^/s water diffusion coefficients). **(B)** (top right) Aqueous RMSF values mapped to CALB structure (Green > 0.10 nm, yellow 0.06–0.10 nm, red < 0.06 nm RMSF values). **(C)** (bottom) Aqueous RMSF averaged over all the ensembles, color coded according to the regional protein hydration shell water diffusion coefficients (Green > 1.5 × 10^−5^ cm^2^/s, yellow 1.0–1.5 × 10^−5^ cm^2^/s, red < 1.0 × 10^−5^ cm^2^/s water diffusion coefficients).

Figure 5A top, left panel and Figure 5B top, right panel show protein hydration shell water diffusion coefficients and RMSF values mapped to CALB structure. According to Figure 5A top, left panel and Figure 5B top, right panel, when we compare water diffusion and RMSF, it can be seen that many regions with fast water dynamics show high flexibilities and regions with slow water dynamics show stability (low RMSF values). This is also seen in the RMSF values per residue (Figure 5C bottom panel), where water diffusion speeds are overlaid in color. A good correlation can be observed between water diffusion and protein flexibilities, with the only regions diverging from *fast solvent* = *flexible region* and *slow solvent* = *rigid region* being the helix α7, which has fast water dynamics but moderate protein flexibility, and the α2 helix and its adjacent loop, which exhibit slow water dynamics but moderate flexibility.

**Figure 6:**
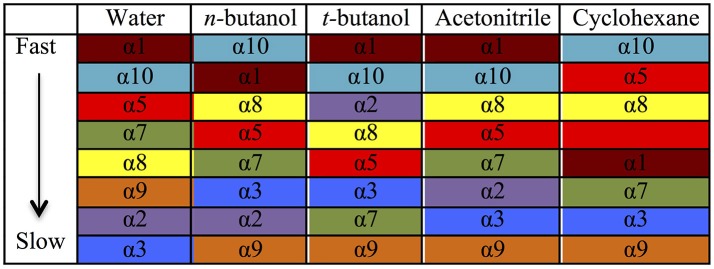
Coefficients of diffusion of each solvent around each α-helix, ranked from fastest to slowest. Each α-helix has its own color to guide the eye to differences among solvents.

Table 2 provides different measures of water dynamics across the protein α-helices. It can be seen that there is a high correlation between the two diffusive measures of water dynamics (residence times and diffusion constants). Generally, fast protein-water hydrogen bond lifetimes are observed in the regions of fastest diffusive motion, but the quantities are not as strongly correlated. Tables 3–6 provide solvent dynamics around each α-helix for the organic solvents *n-*butanol, *tert-*butanol, acetonitrile, and cyclohexane, respectively. As seen with the bulk solvent dynamics (Table 1), the dynamics of the larger solvents are dramatically slower than those of water, particularly butanols' protein-solvent hydrogen bond lifetimes. Furthermore, the regions of the protein surface displaying fast and slow solvent dynamics shift. That is to say, the map of solvent dynamics across the protein surface (like that in Figure 5A for water) depends on the nature of the solvent. Figure 6 also illustrates this, where the regions of protein solvation shells exhibiting fastest to slowest diffusion coefficients are ranked from top to bottom.

In examining the values of solvation layer dynamics in Tables 3–6, it can be seen that some diffusion coefficients of *n-*butanol are close to those of bulk *n-*butanol, while acetonitrile and *tert-*butanol exhibit consistently slowed diffusion constants in local solvation shells, relative to the bulk values (~75% or slower). Tables S2–S5 provide regional solvent shell dynamics in Amber force field, with similar degrees of retardation around exterior protein regions, with the exception of helix α1 in *n-*butanol, which exhibits more hindered dynamics (84% bulk diffusion, vs. 90% bulk diffusion in OPLS-AA) and helix α5 in cyclohexane (79% bulk diffusion in Amber vs. 90% bulk diffusion in OPLS-AA). Notably, diffusion of cyclohexane around the α10 helix is virtually identical to its bulk solvent diffusion coefficient, coinciding with high flexibility of α10 in cyclohexane (see Figure 4, where mobility of α10 in cyclohexane is greater than the mobility of the region in water). Note that residence times of organic solvent are consistently shorter than water residence times, and this arises from the definition of the solvation layer. For water, two layers were considered [out to 5 (7) Å for exterior (interior) regions] in order to have comparison with previously reported values (Denisov and Halle, 1996; Qiu et al., 2006; Huang et al., 2014). For organic solvent, the boundary of the solvation shell is solvent-dependent (see above in Methods), but only the first layer is considered.

**Figure 7:**
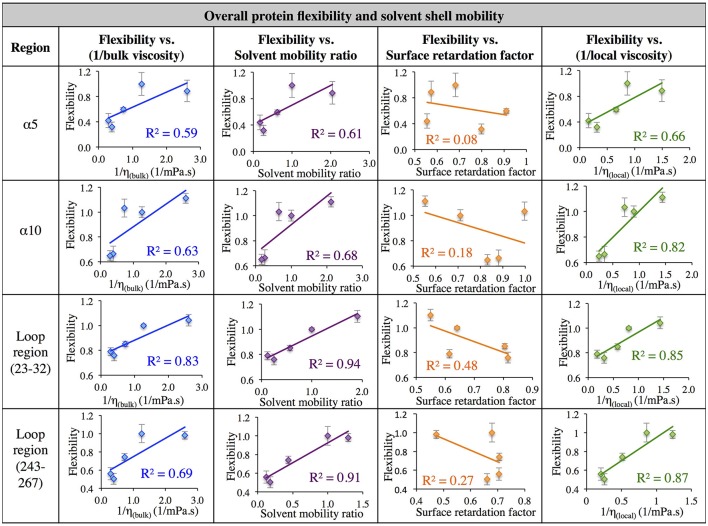
Correlation of flexibility (calculated using all atom RMSF) with bulk viscosity (first column), solvent mobility ratio (second column), surface retardation factor (third column), and local solvent viscosity (fourth column) for the α5 and α10 secondary structures and two loop regions (residues 23–32, and residues 243–267).

Next, in order to further investigate the relationship between solvent dynamics and protein dynamics, correlations between protein flexibility and different descriptors of solvation layer mobility were considered. Protein flexibility was calculated as the protein region's motion in a given solvent, relative to the region's aqueous protein dynamics, so it is described as the ratio of the region's integrated RMSF in organic solvent to the integrated RMSF in the same region in aqueous solvent. Relationships were examined for four different regions: α5, α10, and two loop regions (residues 23–32, and 243–267). The regional flexibility (from all-atom RMSF) is plotted on the y-axis in Figure 7 against four different descriptors of solvent dynamics on the *x-*axis: (1) bulk viscosity (*x* = 1/η_bulk_, _model_); (2) solvent mobility ratio, taking the ratio of diffusion of a given solvent in the regional solvent shell to the diffusion of water in that same region (*x* = D_solvent_, _regional_/D_water, regional_); (3) surface retardation factor, describing how much the solvent slows down in the solvent shell, relative to the bulk (*x* = D_solvent_, _regional_/D_solvent_, _bulk_); (4) local viscosity (*x* = 1/η_local_) from Equation 9, which depends on bulk viscosity and the surface retardation factor. As can be seen in the left column of Figure 7, first a correlation was considered between protein flexibility and bulk solvent viscosity, and a rather poor correlation is seen for most regions, but with enough agreement that it is easy to understand why early models used this bulk solvent property to interpret the dependence of protein dynamics and kinetics on solvent dynamics. The correlation between protein flexibility and solvent mobility ratio (relative mobility of a given solvent in the solvation layer, compared to the native hydration environment) shows improved agreement, indicating that local interactions with the solvent shell influence protein mobility. In fact, for the loop regions, protein flexibility correlates very highly with the ratio of regional solvent diffusion to regional water diffusion at the interface.

Next, the mobility of solvent at the protein interface relative to the solvent's mobility in the bulk was considered (the surface retardation factor), and no correlation was seen with protein flexibility. Finally, a correlation was considered between protein flexibility and local solvation layer viscosity, using Equation 9, and it can be seen that for the α-helices, protein flexibility has the highest correlation with the local solvent viscosity. Recall that bulk viscosity (left-hand side) and local solvation layer viscosity (right-hand side) are not independent variables; rather, the local viscosity is a function of bulk viscosity, related through Equations 3, 9 as $\eta_{local}\approx \;\eta_{bulk}\;\frac{D_{bulk}}{D_{local}},\;$using regional solvation layer (local) diffusion rates. Meanwhile, flexibility in the loop regions is most highly correlated with the relative mobility of organic solvent in the regional solvation layer, relative to water mobility in that same region. In all cases, the results suggest that what really matters with solvent is *location*: the dynamics of the solvation shell surrounding a protein region are correlated with that region's dynamics. It is notable that the lowest correlation between protein flexibility and solvent mobility (any measure) was observed for the region of α5, which is involved in major conformational changes. This is discussed in the next section.

**Figure 8:**
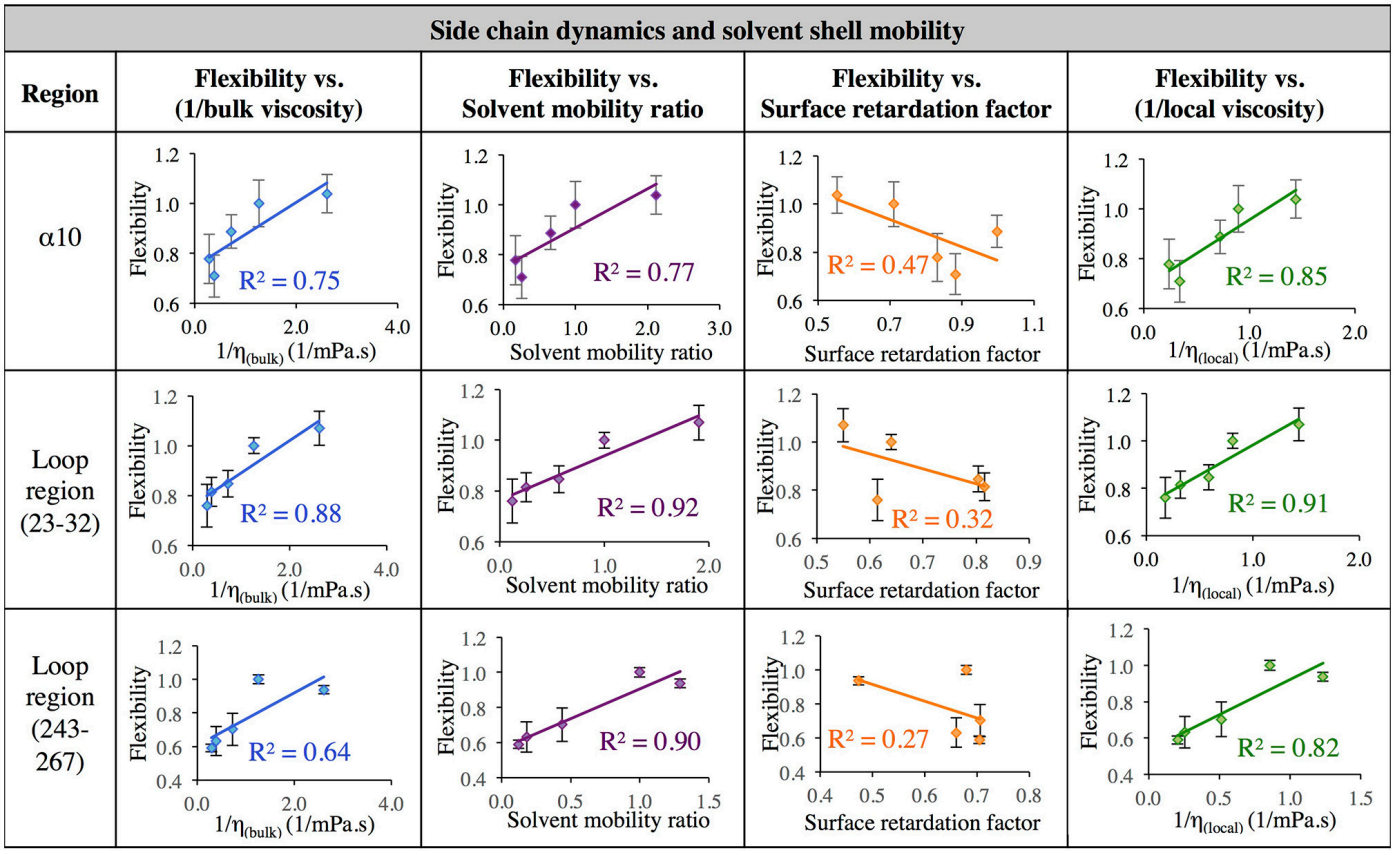
Correlation of flexibility (calculated using protein side chain RMSF, relative to water RMSF) with bulk viscosity (first column), solvent mobility ratio (second column), surface retardation factor (third column), and local solvent viscosity (fourth column) for the α10 secondary structure and two loop regions (residues 23–32, and residues 243–267).

**Figure 9:**
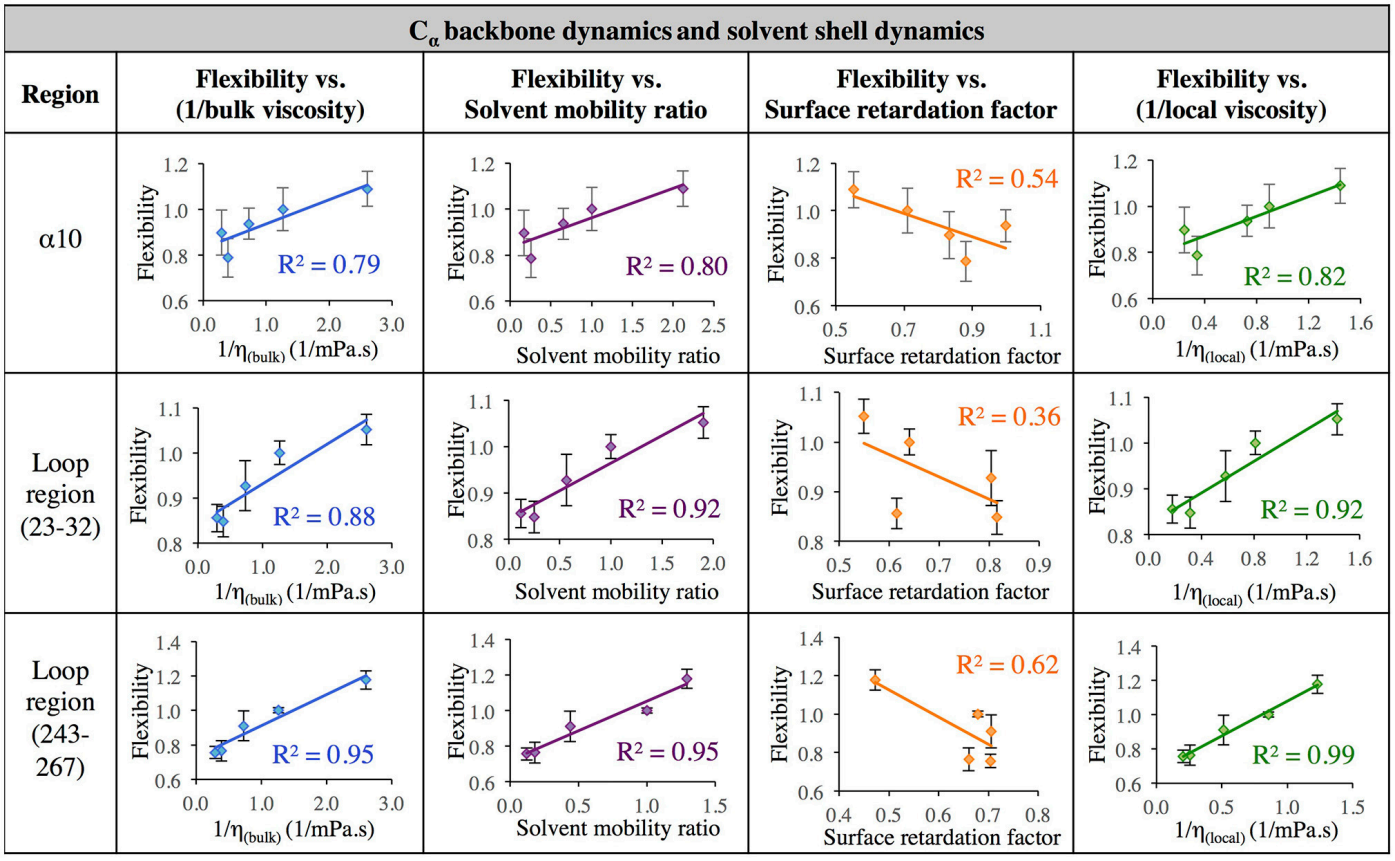
Correlation of flexibility (calculated using protein C_α_ RMSF) with bulk viscosity (first column), solvent mobility ratio (second column), surface retardation factor (third column), and local solvent viscosity (fourth column) for the α10 secondary structure and two loop regions (residues 23–32, and residues 243–267).

The data in Figure 7 evaluates the dynamics of both the protein backbone and side chains via total RMSF value. They raise a question of whether local solvation layer mobility has a larger effect on side chain dynamics or backbone dynamics. To evaluate this, the RMSF values of side chains (only) and the protein backbone (C_α_) alone were considered. Analysis of the correlation between different descriptors of solvation layer mobility (akin to those in Figure 7) and protein dynamics were considered for side chains only (Figure 8) and backbone dynamics (Figure 9) for α10, and two loop regions (residues 23–32 and 243–267), where the highest correlations between total protein flexibility and solvent mobility were observed (Figure 7). In Figure 8, protein flexibilities were calculated as the ratio of the region's integrated side chain RMSF in organic solvent vs. aqueous solvent. The same analysis was done for backbone flexibility in Figure 9. Separating the motions (which are known to take place on different timescales: side chains moving picoseconds-to-nanoseconds and backbone dynamics in the nanoseconds-to-microseconds regime) provides some insight. The backbone motion of all three regions evaluated has the highest correlation with the local viscosity (relative to any other measures of solvent mobility), with loop 243–267 remarkably having a correlation coefficient of 0.99 for 1/η_local_. For side chain flexibility, however, the highest correlation for the dynamics of the loop regions is with the solvent mobility ratio, which compares the mobility of organic solvent to that of water at the interface. Meanwhile, the side chain dynamics of the α10 region has the highest correlation with (inverse) local viscosity, as seen for the backbone.

**Figure 10:**
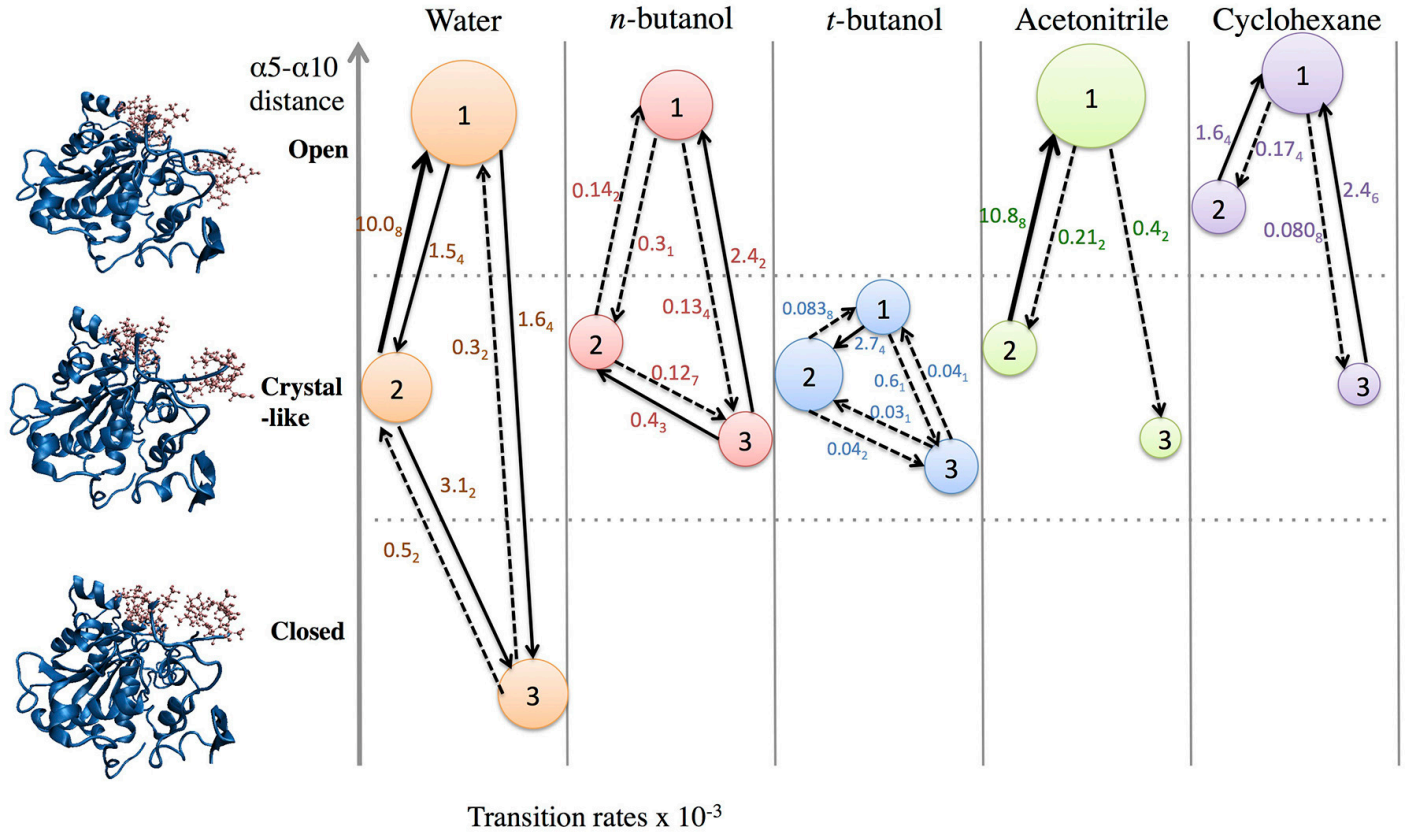
Transition rates (x 10^−3^ ns^−1^) between conformational states, according to hidden Markov state model (uncertainties in last digit are given as subscript). The sizes of circles around states 1, 2, 3 indicate relative populations of each conformation. The fastest transitions are given in solid arrows; dashed arrows represent slow or infrequent conformational transitions.

In order to get statistical analysis of differences in protein dynamics, conformational transition rates were obtained using hidden Markov state model. Figure 10 shows transition rates between metastable (long-lived) states, which are distributed among the conformational states of CALB enzyme (Figure 3), characterized by the α5–α10 distance. It can be seen that the fastest transition rates were observed in the presence of water and acetonitrile solvents for the crystal-like to open conformational changes. This is in accordance with the high flexibility of the α5 region indicated in the RMSF, Figure 4, in the presence of both water and acetonitrile. The transition from crystallographic to open conformations occurs in both water and acetonitrile with a probability of ~10 (× 10^−3^ ns^−1^). Further, it should be noted that in the presence of these two solvents, fast solvent dynamics were observed around the α5 region, confirming the correlation of fast solvent dynamics around α5 with high protein flexibility and fast conformational changes. In contrast, the rates of conformational changes are much slower in the larger, less polar organic solvents. Cleft opening motions are slowed to varying degrees: ~2 (× 10^−3^ ns^−1^) in cyclohexane and *n-*butanol, 0.04–0.08 (× 10^−3^ ns^−1^) in *tert-*butanol. Cleft closing motions are fastest in water (1–3 × 10^−3^ ns^−1^), and an order of magnitude slower in organic solvent. In the presence of all solvents, except for *tert*-butanol, the highest conformational transition rate was observed for the crystal-like to open conformational changes. In *tert*-butanol, all three metastable states were distributed in crystal-like conformation only. The data in Figure 10 indicates that the underlying potential energy surface governing open/crystallographic/closed conformations is shifted, with organic solvents favoring the open conformation (as seen previously by Trodler and Pleiss, 2008; Li et al., 2010; Ganjalikhany et al., 2012).

## Discussion

The flexibility of most regions of CALB in water is seen in Figure 5 to correlate with local hydration layer mobility, which makes sense physically. Since protein dynamics are Brownian (thermal) motion, their diffusive motions depend on friction arising in the surrounding solvent environment. More mobile solvation layers should promote faster (and farther) protein motions, enhancing flexibility. A more static solvent cage, meanwhile, should promote protein stability by hindering protein motions. A notable result in the heterogeneous dynamics of CALB's hydration shell (i.e., in its native solvent), is the fast waters around the α5 helix, which moves for substrate binding and release and whose dynamics are rate-contributing (Uppenberg et al., 1994; Zisis et al., 2015). Water has a very short hydrogen bond lifetime with the protein and fast diffusion around this region, making one wonder whether the enzyme evolved fast regional hydration layer dynamics to promote enzyme dynamics-function. The results for aqueous CALB showing correlations between regional hydration dynamics and protein structural flexibility/stability raise the question whether this is a general motif seen in protein hydration dynamics, when mapped around the solvent-exposed surface and compared alongside protein dynamics (which can be analyzed through NMR spectroscopic data, structural B-factors, RMSF values, or conformational transition rates). Site-specific coupling between protein and solvent dynamics has been observed spectroscopically (King et al., 2012; Qin et al., 2016) and in simulations (Ebbinghaus et al., 2007) (Oroguchi and Nakasako, 2016). Data for CALB in organic solvent supports a dependence of protein dynamics on regional solvation layer dynamics, with regions of high solvent mobility correlating to protein flexibility (such as the α10 region of CALB in cyclohexane) and low solvent mobility correlating to hindered protein dynamics (such as the α5 region in *n-*butanol).

In aqueous solutions of glycerol, ethanol and ethylene glycol, Beece et al. found protein dynamics, and conformational transition rates, to be inversely proportional to solution viscosity, η (with deviations at high viscosity for heme). Their results, reported in 1980, provided groundbreaking insight into protein dynamics and the connection between solvent and protein dynamics (Beece et al., 1980). Our data indicates that for CALB in non-aqueous solvent, the relationship between regional protein dynamics and 1/η is not completely sufficient to describe certain protein motions (Figures 7–9, first column). We are proposing that effects from *local* solvent dynamics, within the solvation shell, provide more physical insight than bulk viscosity alone. Our results in Figures 7–9 support this, with high correlations observed between local solvent shell mobility and protein flexibility (fourth column) in the α10 helix and loop regions 23–32 and 243–267.

The data presented in results suggests that comparison of local water mobility with local mobility of other solvents will predict solvent effects on fast side chain dynamics. The structural dynamics, or backbone motions, are well-predicted by the “local viscosity,” which is the product of bulk solvent viscosity and the ratio of bulk diffusion to regional, interfacial diffusion (Equation 9). A more formal theoretical treatment, such as explicit calculation of friction or use of mode coupling theory (Götze, 1998), may be more rigorously correct. However, calculation of local viscosity or solvent shell mobility from local measures of solvent dynamics, as we are suggesting here, can be carried out spectroscopically and may be a sufficiently quantitative and tractable approach. The model can be readily tested by comparing solvation shell dynamics at different locations, which can be achieved through a variety of spectroscopic techniques (e.g., ultrafast fluorescence, IR, NMR spectroscopies). Using simulations to predict changes in solvation shell dynamics is also viable, as calculated solvent dynamics have reasonably reproduced experimental measurements in recent studies (King et al., 2012; Dielmann-Gessner et al., 2014; Abel et al., 2016; George et al., 2016; Charkhesht et al., 2018).

With foresight regarding which protein regions one is wishing to be stabilized or given enhanced flexibility, solvent shell dynamics could be engineered through point mutations to yield improvements in biocatalysts in desired solvents, or to tune the stability or function of engineered proteins. For example, in regions where protein mobility influences enzyme kinetics, organic solvent shell dynamics could be characterized and compared to water dynamics in the same region. If the solvent shell mobility (or local viscosity) is significantly different from that of water's, protein mutations to speed up solvent in the region to enhance protein flexibility could be targeted. In a similar vein, if an organic solvent destabilizes protein structure, it may be possible to stabilize the protein by slowing down the solvent shell around regions requiring more stability, and NMR analysis may be effective in identifying regions where protein unfolding first takes place (Roder et al., 1988; Udgaonkar and Baldwin, 1988; Frieden et al., 1993). Effects of mutations on coupled solvent-protein dynamics could be monitored spectroscopically (or with MD analysis similar to work undertaken here). Protein dynamics have a wide range of time scales, ranging from picoseconds and nanoseconds time scale for local flexibility (methyl rotations, side chain rotations, loop motions) to microseconds and milliseconds time scale for collective motions (larger domain motions). Different experimental studies have been carried out to examine these protein motions at different time scales. For example, NMR spectroscopy is used to study many dynamical processes in proteins (Wand, 2001; Kempf and Loria, 2003; Boehr et al., 2010; Caro et al., 2017), since site-specific information can be obtained for dynamics over a wide range of time scales (Mittermaier and Kay, 2006). In NMR studies, protein dynamics in the nanosecond and picosecond time scale are referred to as fast dynamics and are detected mainly through relaxation rates, whereas protein dynamics in the microsecond or larger time scale are referred to as intermediate to slow dynamics, and are quantified using line shape analyses, rotating frame relaxation rates, magnetization transfer, and selective inversion recovery methods (Palmer et al., 1996). Recent work has observed fast protein motions using cross-related ^1^H-^1^H NMR spin relaxation in methyl groups (Capdevila et al., 2017). An intriguing use of NMR spectroscopy to evaluate protein dynamics is through the use of fluorolabelled amino acids. Hoeltzli and Frieden observed conformational changes in dihydrofolate reductase using ^19^F NMR spectroscopy (Hoeltzli and Frieden, 1998). The extreme sensitivity of ^19^F resonance to its non-bonded environment leads ^19^F NMR to provide details regarding protein conformational changes with rapid data acquisition and no heavy isotope labeling (Dahanayake et al., 2018).

In other spectroscopic studies, analysis of fast protein dynamics with various solvents using Raman and neutron scattering, strong coupling between fast picosecond protein dynamics and surrounding solvent dynamics is observed. In contrast, NMR and simulations data indicate that methyl group rotations are more independent from hydration water (Mittermaier and Kay, 2006; Krishnan et al., 2008). Another useful spectroscopic technique for analyzing effects of mutations on solvent and protein dynamics is tryptophan fluorescence spectroscopy, which is used to report on both solvent dynamics and protein side chain dynamics. For example, using tryptophan scanning with femtosecond fluorescence spectroscopy, Zhong and co-workers measured both hydration shell water relaxation and protein side-chain fluctuations. The dynamical processes of protein and solvent shell were seen to have the same energy barriers, suggesting that hydration shell dynamics drive protein side-chain fluctuations, although hydration water dynamics were observed to be faster than protein side-chain relaxations (Qin et al., 2016).

Although spectroscopic techniques can evaluate effects of mutations on solvent and protein dynamics, without a systematic understanding of how protein structure influences the dynamics of various organic solvents, the effects of mutations on regional solvent shell dynamics would need to be modeled to screen for mutations that bring about desired changes. We have recently used multi regression analysis to evaluate how protein topology and hydrophobicity influence water structure and dynamics in the hydration layer around CALB, and have seen that protein surface topology (i.e., convex, flat, concave regions) accounts for ~75% of effects on water density in the hydration shell, while hydrophobicity accounts for the remaining 25%. The density of the hydration layer, in turn, is strongly correlated with water dynamics, which is an entropic effect. Work is underway to carry out similar analysis of solvent shell structure-dynamics relationships for organic solvents, while looking at correlations with various properties of the protein surface structure (Dahanayake and Mitchell-Koch, 2018).

Analysis suggests that mutations at non-conserved sites often slightly modulate function rather than drastically influencing structure-dynamics-function (Meinhardt et al., 2013). Furthermore, it was recently observed that a single protein mutation causes measurable alterations in the collective water dynamics in green algae (Russo et al., 2016). Solvent selection could also be aided by mapping the dynamics of various organic solvents to the protein surface, and selecting those with predicted dynamics that are compatible with desired regions of flexibility and stability (or perhaps with patterns of fast and slow dynamics that are most similar to water at key regions).

### Comments, caveats, and questions

- Do the dynamics of aqueous solutions (mixed organic/aqueous solvents) in solvation shells resemble those of pure water in solvation shells? If so, this may contribute to the success of previously formulated models, such as solvent slaving, describing relationships between temperature-dependent **bulk** solvent properties of *aqueous solutions*, such as viscosity and dielectric relaxation rates, and protein dynamics/transition rates. The temperature-dependence of regional solvation shell dynamics is an area of active investigation, and we anticipate that investigation of more localized solvent layer dynamics may provide more insight.
- Local solvation shell dynamics are a function of bulk solvent dynamics (scaled to a certain degree by interactions with the protein structure). The coefficient *n(T)* in Equation 2 (describing solvent slaving, Fenimore et al., 2002) could be revisited to explicitly account for solvation shell retardation, with n(T) depending on protein region, nature of the solvent, and the specific ways in which a protein modulates a solvent's dynamics. Figure 6 and Tables 2–4 in the results section clearly indicate that the influence of each protein region on solvent shell dynamics differs by solvent, presumably depending on solvent polarity, size, shape, and hydrogen-bonding characteristics.
- The high correlation between the solvent mobility ratio (diffusion of an organic solvent relative to that of water in the region) and protein flexibility (particularly side chain motions and the backbone dynamics of loop regions) may provide insight into the role of water in protein function. The local hydration shell provides a native environment, with a given amount of friction that arbitrates the local dynamics and flexibility of the protein. The data in Figures 7–9 suggests that solvent that moves similarly to interfacial water maintains the native protein dynamics.
- Our previous work addressed the question of keeping (or not keeping) crystallographic water molecules in simulations of enzymes, and we found that slowly-diffusing waters in organic solvent should be kept in order for simulations to quickly equilibrate (as described in Methods). We also saw that the location of retained waters affects conformational sampling (Dahanayake et al., 2016). We have postulated that both buried and slow-diffusing water molecules are the ones which are retained experimentally when enzymes are used in organic solvent. When preparing CALB in organic solvent for this work, seven slow-diffusing water molecules around the enzyme were observed. Among those seven water molecules, two are buried in the interior of CALB, one of which is hydrogen bonded to Asp 187, a catalytic triad residue. However, five of the slow-diffusing water molecules are located in the solvation layer around the exterior of the protein. These waters have hydrogen bonds with exterior residues (residues 20–22, 58, 96, 124, and the β9 region). These observations emphasize that slow-diffusing water molecules in the first hydration shell may be considered an integral part of the protein, which should be kept in mind when evaluating the dynamics of solvent shells.
- Particularly in organic solvents and other non-aqueous systems, the protein's underlying conformational potential energy surface may be rather dramatically altered by solvent-protein interactions (ΔG_solv_) (Zhao et al., 2013). The results in Figures 3, **10** suggest this, as open conformations of CALB are favored in organic solvent, in agreement with previous reports (Trodler and Pleiss, 2008; Li et al., 2010; Ganjalikhany et al., 2012). We had hoped that Markov modeling would provide more insight into how solvents affect the timescales of conformational changes in CALB, but found instead that direct comparisons could not be made due to solvent-induced differences in conformational equilibria. One way that solvent can alter the protein conformational equilbria, or the underlying potential energy surface, is by strengthening intraprotein H-bonds and salt bridges as solvent dielectric decreases (Affleck et al., 1992). We examined intraprotein hydrogen bond lifetimes in this data set, and did not see significant differences among solvents for this protein, but do not necessarily anticipate this is a general observation.
- The data in Figures 7–9 suggest that a model based on local viscosity or solvent mobility ratio (when compared to regional hydration dynamics) may accurately predict solvent effects on regional protein flexibility, with transition rates between conformations presumably described by Kramers' theory. For example, excellent correlation between local viscosity in all solvents and relative RMSF of the backbone is observed for the loop regions (Figure 9), which may indicate that the underlying potential energy surface governing the motion of the loops is similar across all the solvents considered. We would propose that hypotheses could be tested and refined by examining the solvent-dependence of H^*^, then using Kramers' theory for conformational transition rates, *k*_*protein region*_, as follows:
  (10) $$\begin{array}{r} k_{protein\;region}\propto \;\frac{1}{\eta_{local}}e^{\frac{-H,solvent*}{RT}} \end{array}$$
  where η_*local*_ is the local solvation layer viscosity (i.e., in the solvent shell surrounding the protein structure involved in dynamics), given by $\eta_{local}\approx \;\eta_{bulk}\;\frac{\tau_{2,\;local}}{\tau_{bulk}}$ or $\eta_{local}\approx \;\eta_{bulk}\;\frac{D_{bulk}}{D_{local}}$ from regional (spectroscopic) measures of solvation layer dynamics, and H,solvent^*^ is the dynamical barrier determined for the protein motion within a given solvent. This latter quanitity could be obtained in MD simulations with constrained distances (den Otter and Briels, 1998) or umbrella sampling. This would provide a link between protein structural dynamics and enzymatic rates for processes where conformational changes are rate-contributing.
- For many of these questions, investigations in sets of homologous solvents may yield further insight.

## Conclusions

In the protein simulations community, it is acknowledged that using implicit solvent or TIP3P water model, which has faster diffusion and lower viscosity than real water, enhances sampling of the protein conformational landscape (Braun et al., 2014; Palazzesi et al., 2016). What if enzyme structures leverage a similar strategy, by maintaining high (bulk-like) mobility in solvation shell waters around regions where more flexibility is required? The hydration dynamics around the CALB surface were shown in our simulations to be heterogeneous. Furthermore, the regional flexibilities of the enzyme were shown to correlate with the regional dynamics of the hydration layer. That is to say, mobile or flexible regions of the protein have fast-moving waters, while stable or less mobile regions generally are surrounded by slower hydration layer water molecules. The connection between solvent shell dynamics and protein dynamics extends into the data obtained for CALB in four organic solvents: acetonitrile, cyclohexane, *n-*butanol, and *tert-*butanol. Regions of CALB (e.g., helix α5) that are flexible in water, but have slow *local* solvent shell dynamics in certain organic solvents, are shown to have hindered structural dynamics in those solvents. Meanwhile, when fast solvent dynamics arise in the solvation layer, CALB can display higher regional flexibility in organic solvent than in water (e.g., helix α10 in cyclohexane).

We propose that future work explore this connection further by using Kramers' or Grote-Hynes theory (Grote and Hynes, 1980) to model protein dynamical transitions, taking into account the local friction (directly calculated, or based on measurements of dynamics in the local solvation layer) and the solvent-dependence of the transition barrier height. If the relationship holds, then solvation shell engineering could be used for biocatalysts and *de novo* proteins to enhance protein stability or flexibility (and dynamics-dependent enzymatic rates) in desired regions. Our recent work on the hydration shell of CALB suggests solvent shell *structure* determines its dynamics, which may aid a rational approach to tuning regional hydration dynamics around biomolecules (Dahanayake and Mitchell-Koch, 2018). Work is underway to see if the same relationships hold for organic solvent. Understanding structure-dynamics connections among enzymes and their solvation shells holds promise to unlock enzymes—both figuratively and literally, in freeing up enzymes' motions– for expanded use in organic solvents and other non-traditional solvents, such as ionic liquids.

## Author contributions

JD carried out and designed research and analysis and wrote article. KM-K designed research and analysis; wrote article.

### Conflict of interest statement

The authors declare that the research was conducted in the absence of any commercial or financial relationships that could be construed as a potential conflict of interest.
