# Supplementary material for: How Does Solvation Layer Mobility Affect Protein Structural Dynamics?
Source: Front Mol Biosci. 2018 Jul 13;5:65. doi: 10.3389/fmolb.2018.00065 (PMC6053501; doi:10.3389/fmolb.2018.00065)
Supplement: Supplementary file 1 [file Data_Sheet_1.docx]

***Supplementary Material***

**How does solvation layer mobility affect protein structural dynamics?**

Jayangika N. Dahanayake^1^ and Katie R. Mitchell-Koch^1^*

*Correspondence: Katie R. Mitchell-Koch ([katie.mitchell-koch@wichita.edu](mailto:katie.mitchell-koch@wichita.edu))


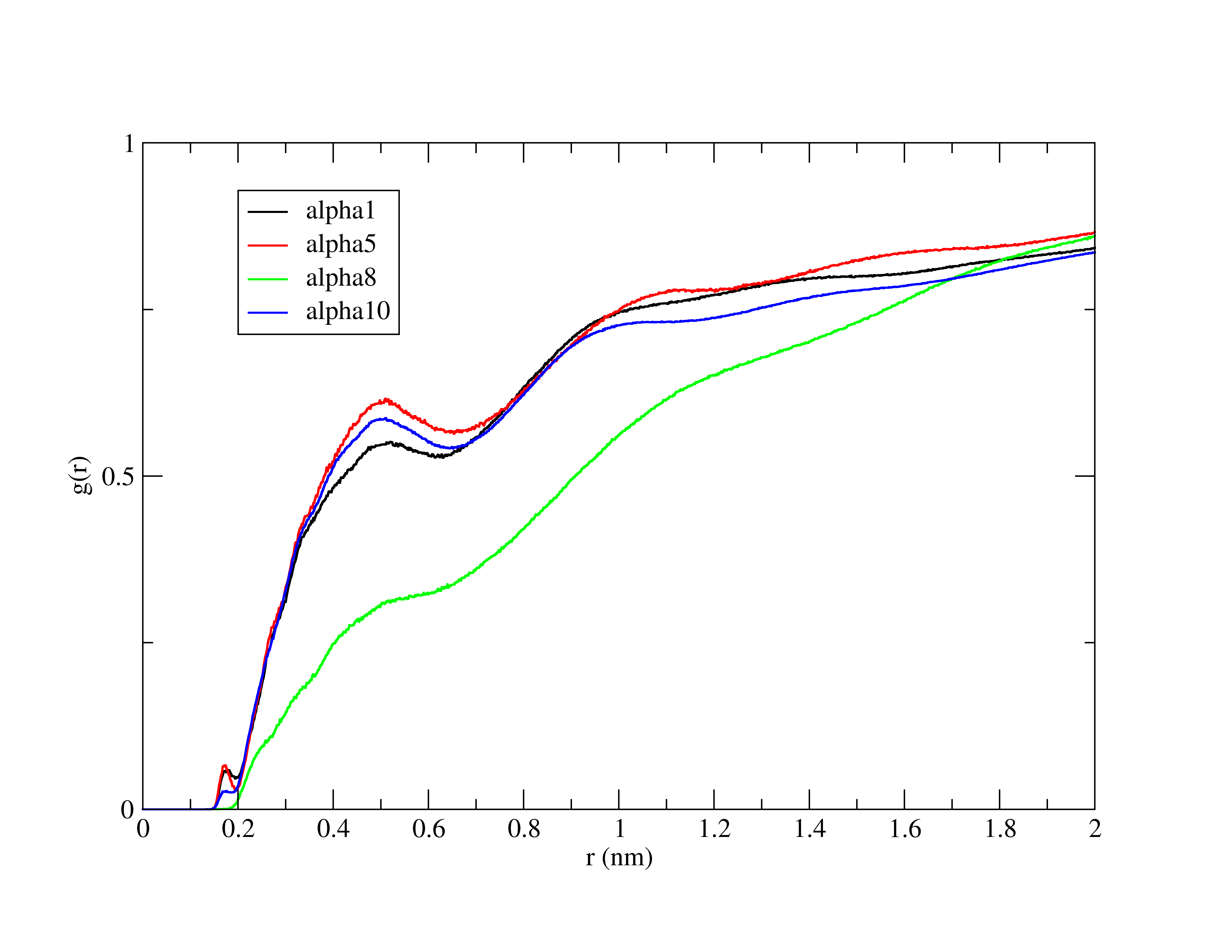


(a) RDF of *n-*butanol around side chain


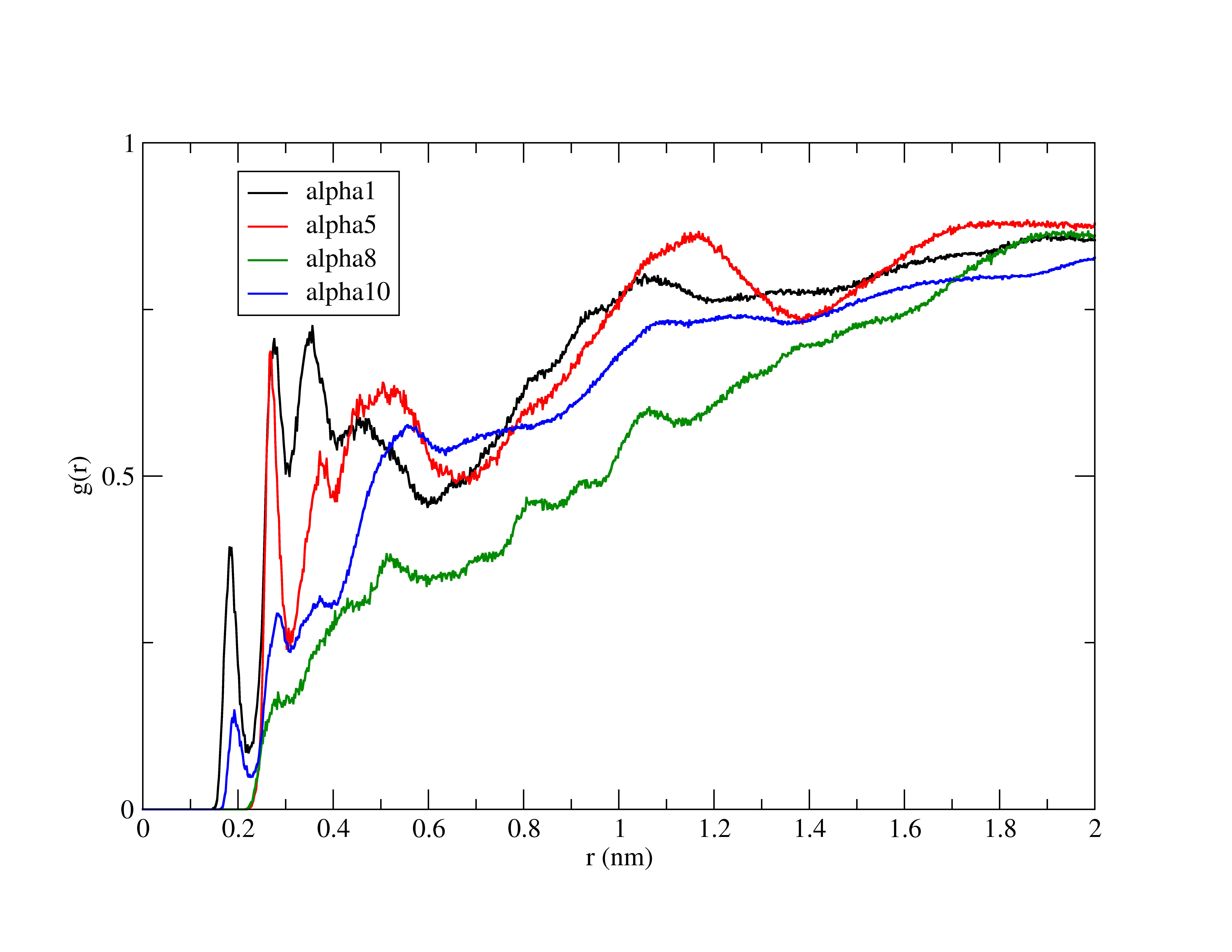


(b) RDF of *tert-*butanol around side chain


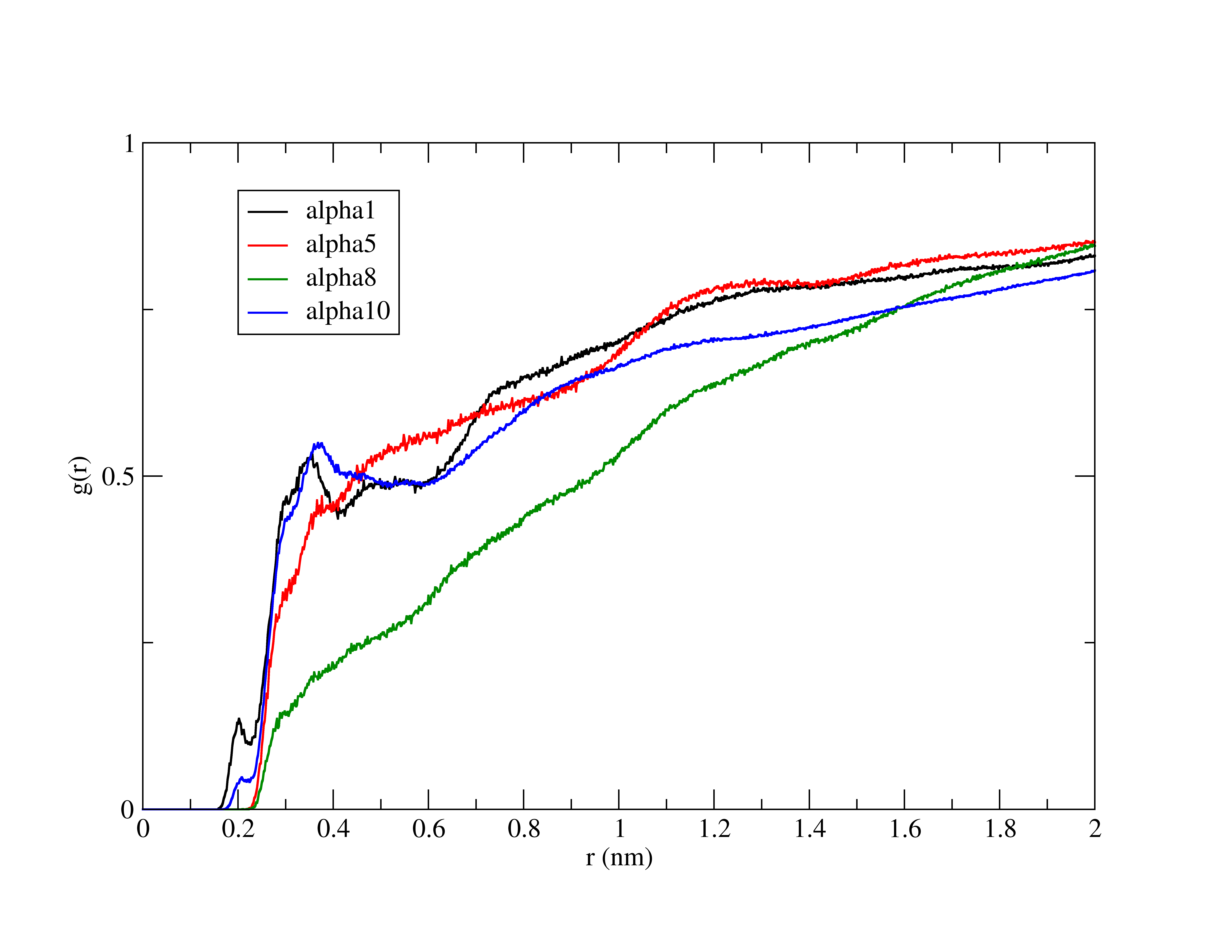


(c) RDF of acetonitrile around side chain


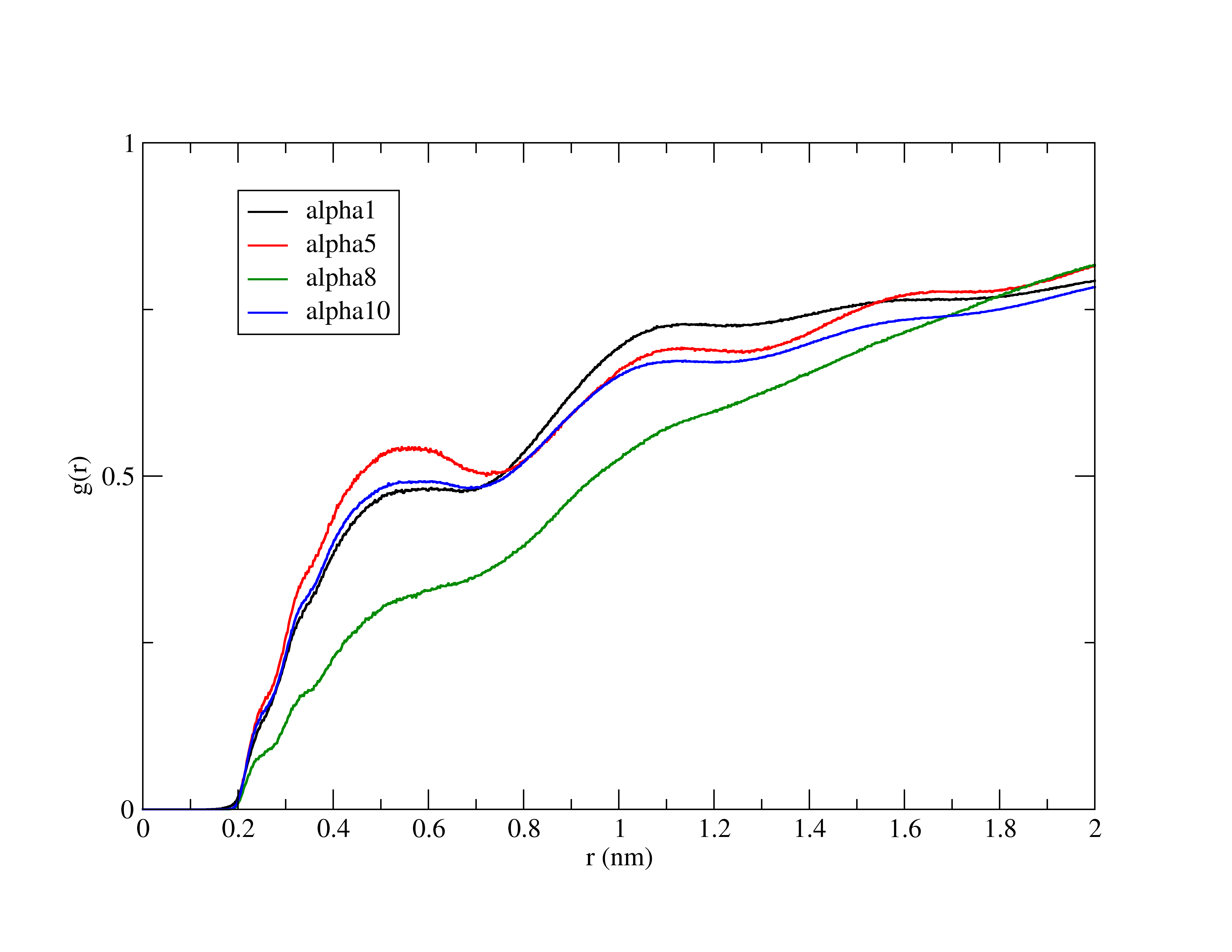


(d) RDF of cyclohexane around side chain

Figure S1. Radial distribution functions of (a) *n-*butabol, (b) *tert-*butanol, (c) acetonitrile, and (d) cyclohexane around side chain atoms of exterior alpha helices. Black indicates α1, red indicates α5, green indicates α8, and blue indicates α10 helices.


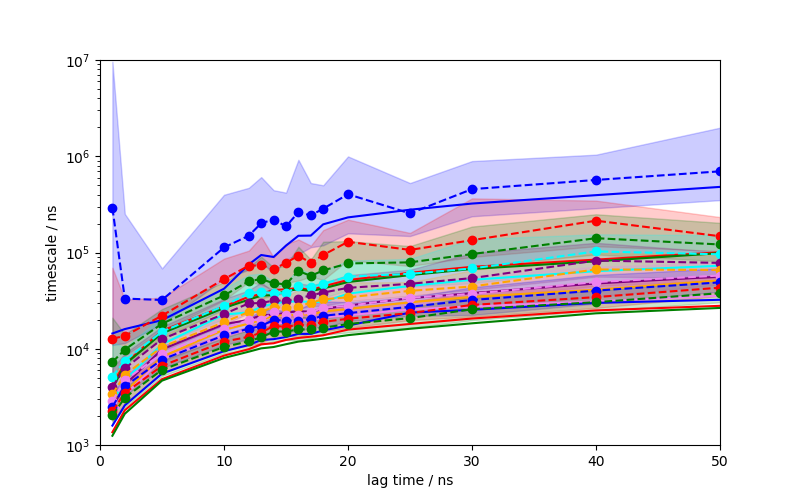


(a)


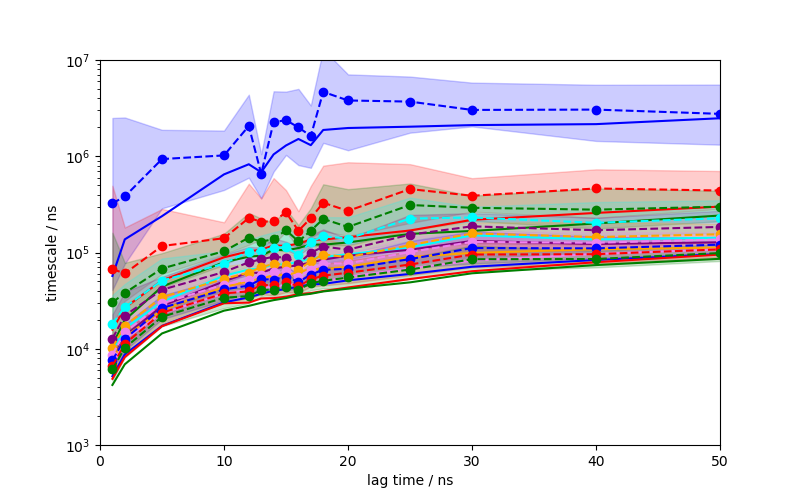

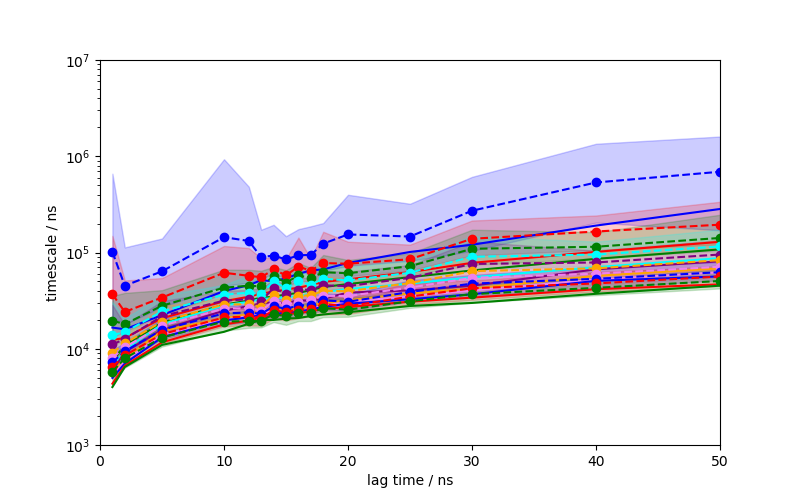

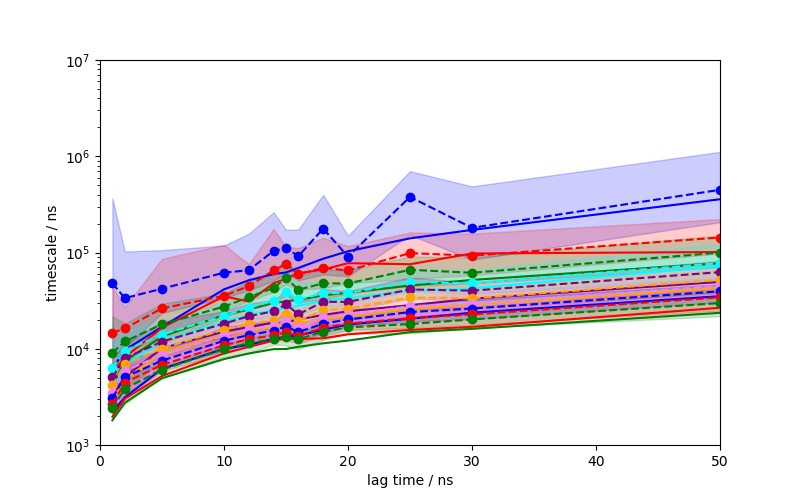

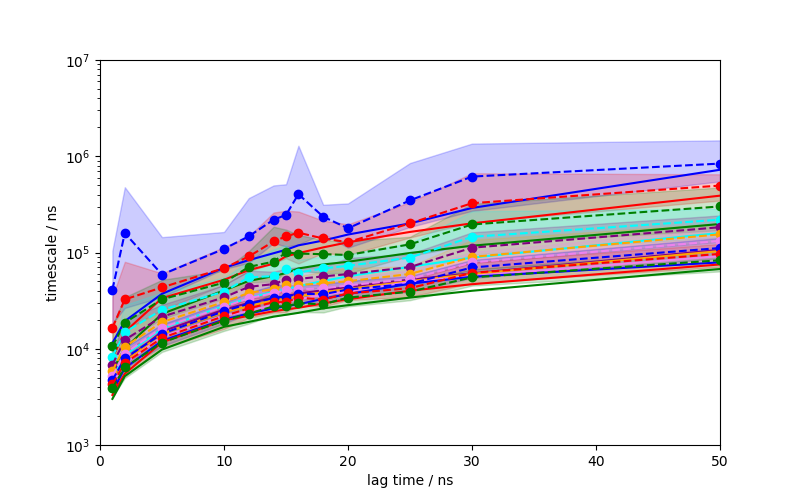


(d)

(c)

(b)

(e)

Figure S2. Implied timescales at a series of lagtimes for (a) water, (b) *n-*butanol, (c) *tert-*butanol, (d) acetonitrile, and (e) cyclohexane.


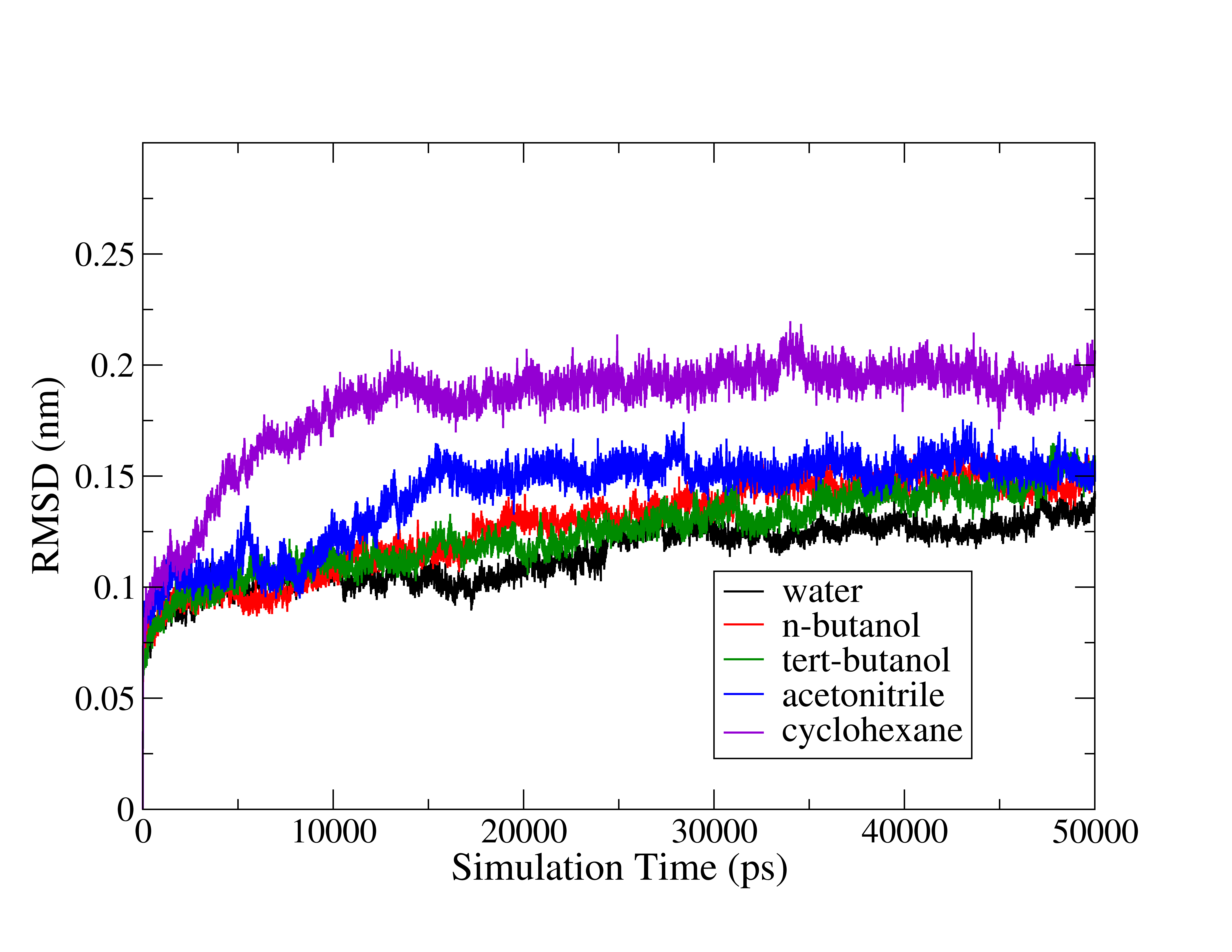

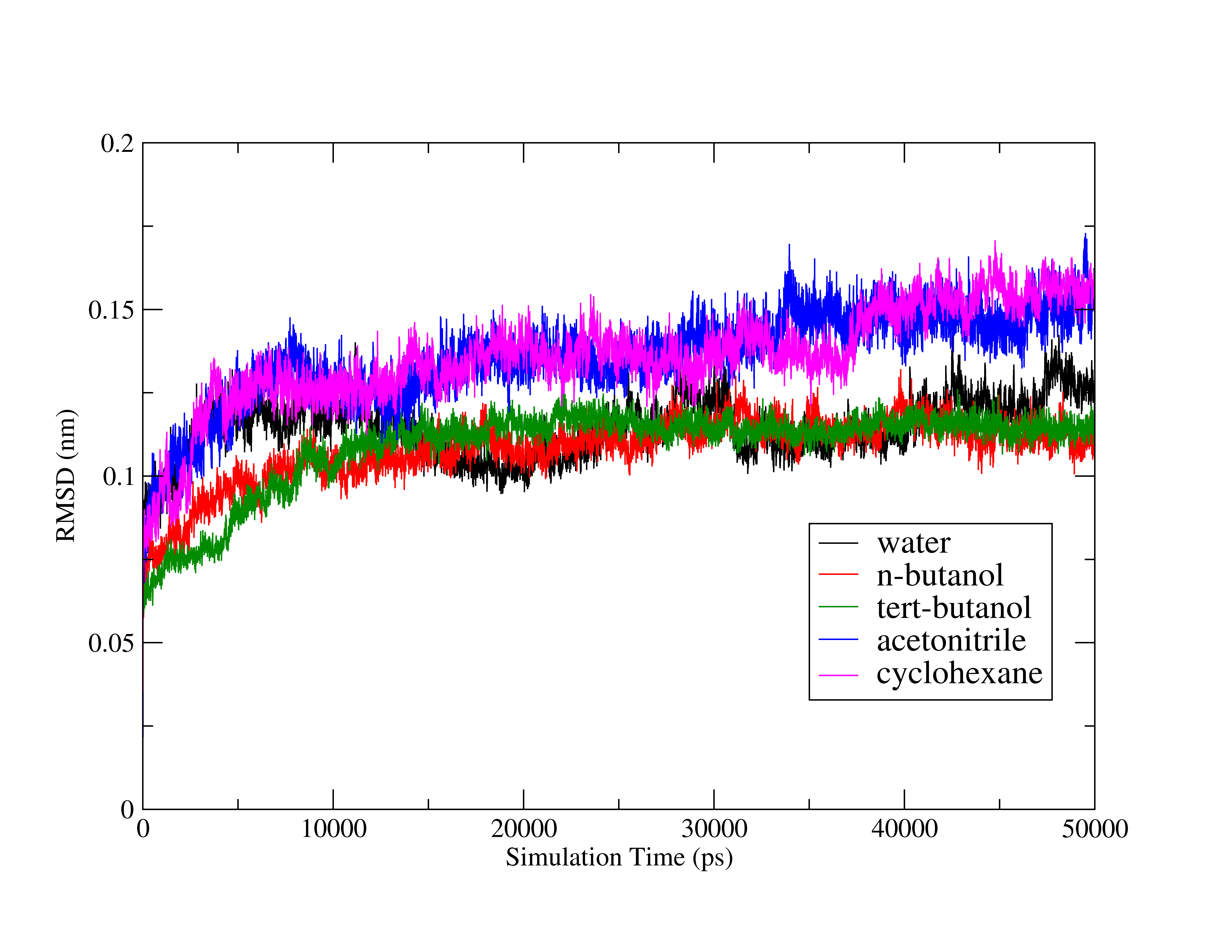


(a)

(b)

Figure S3. RMSD of CALB all atoms as a function of time in the solvents studied with (a) OPLSAA forcefield (b) AMBER forcefield.

Figure S4. Average RMSD (relative to crystal structure) of the all atoms of CALB in the organic solvents calculated from the last 1 ns of six trajectories.

Figure S6. RMSF (obtained using AMBER forcefield) comparison between each organic solvent studied and water (black trace): (a) red- *n-*butanol, (b) blue- *tert-*butanol, (c) green- acetonitrile, and (d) magenta- cyclohexane.

(d)

(c)


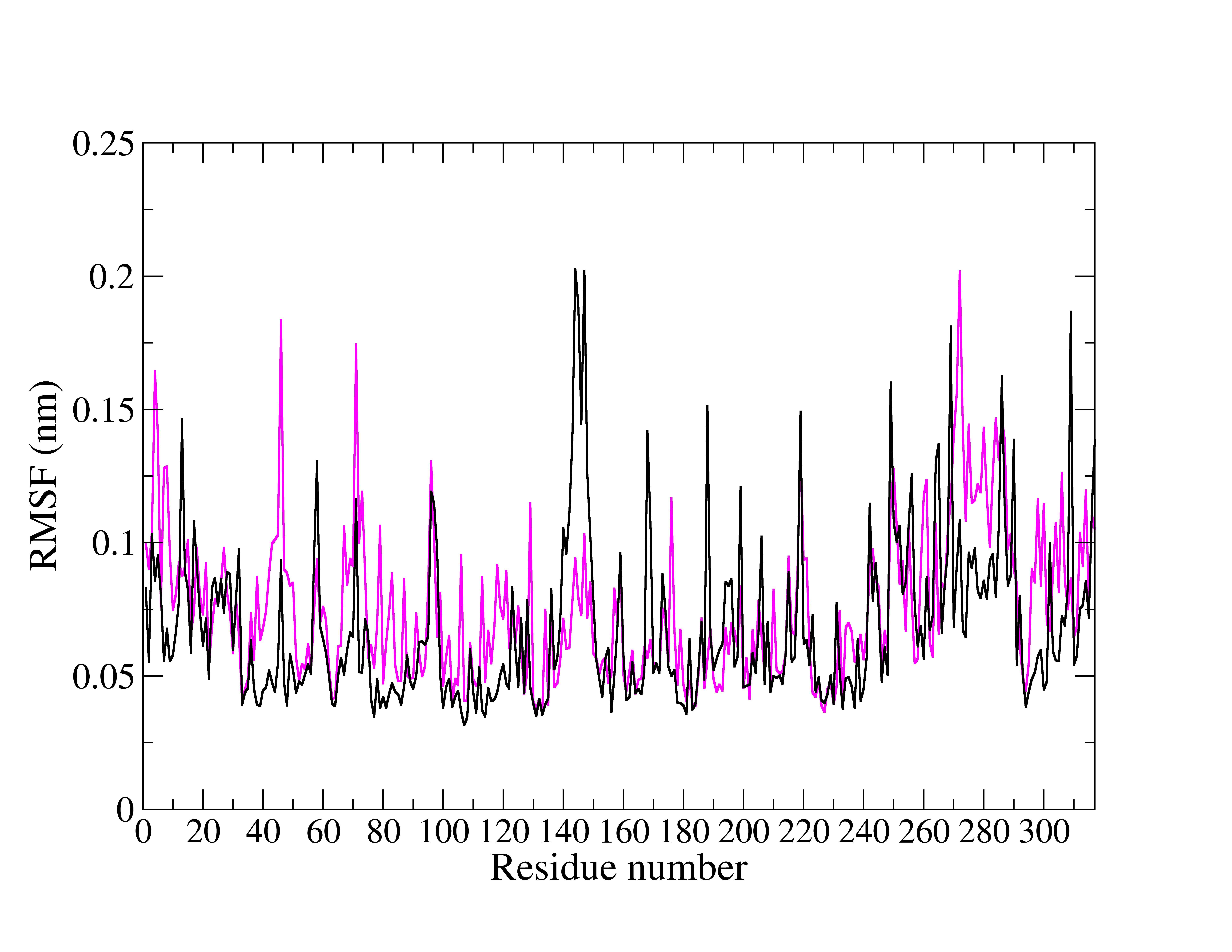

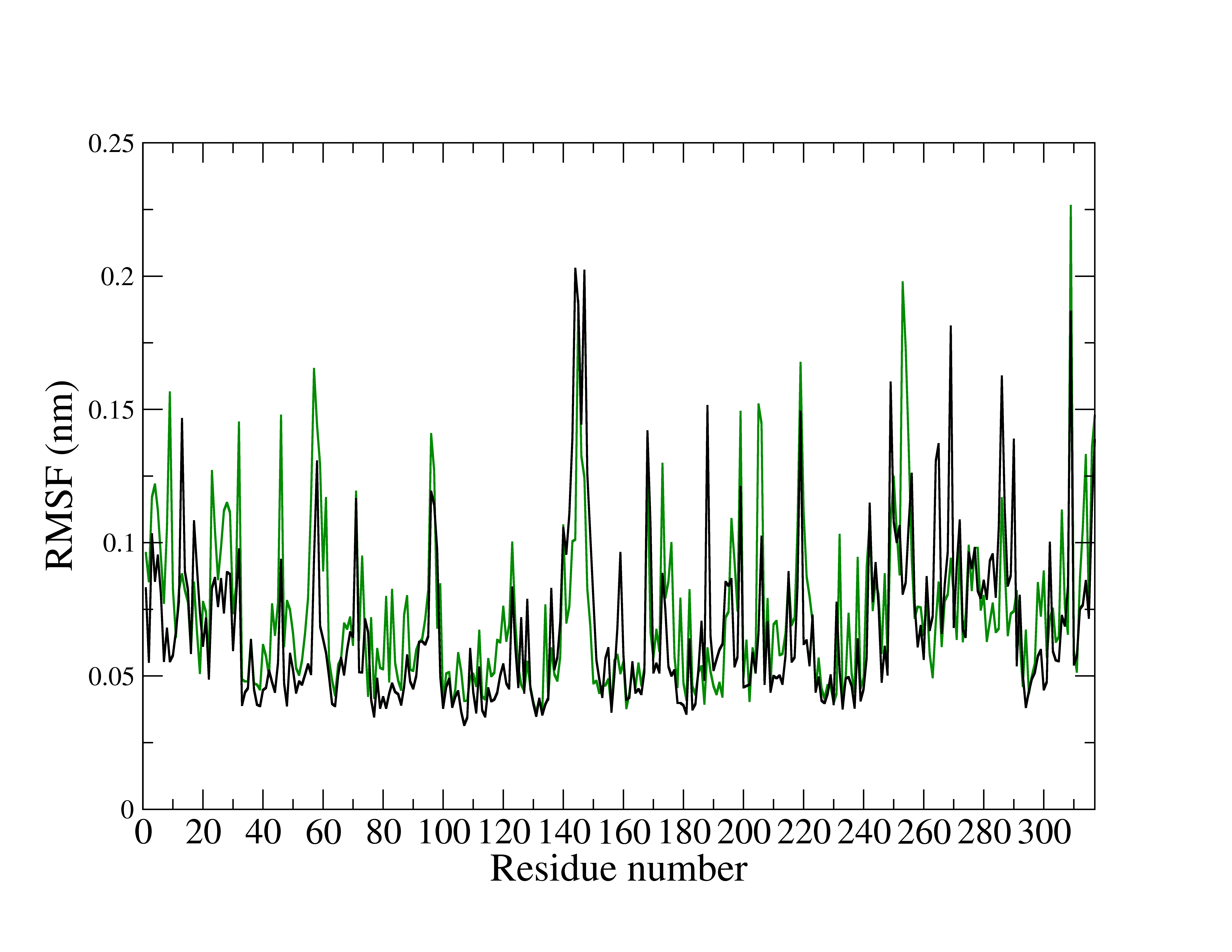

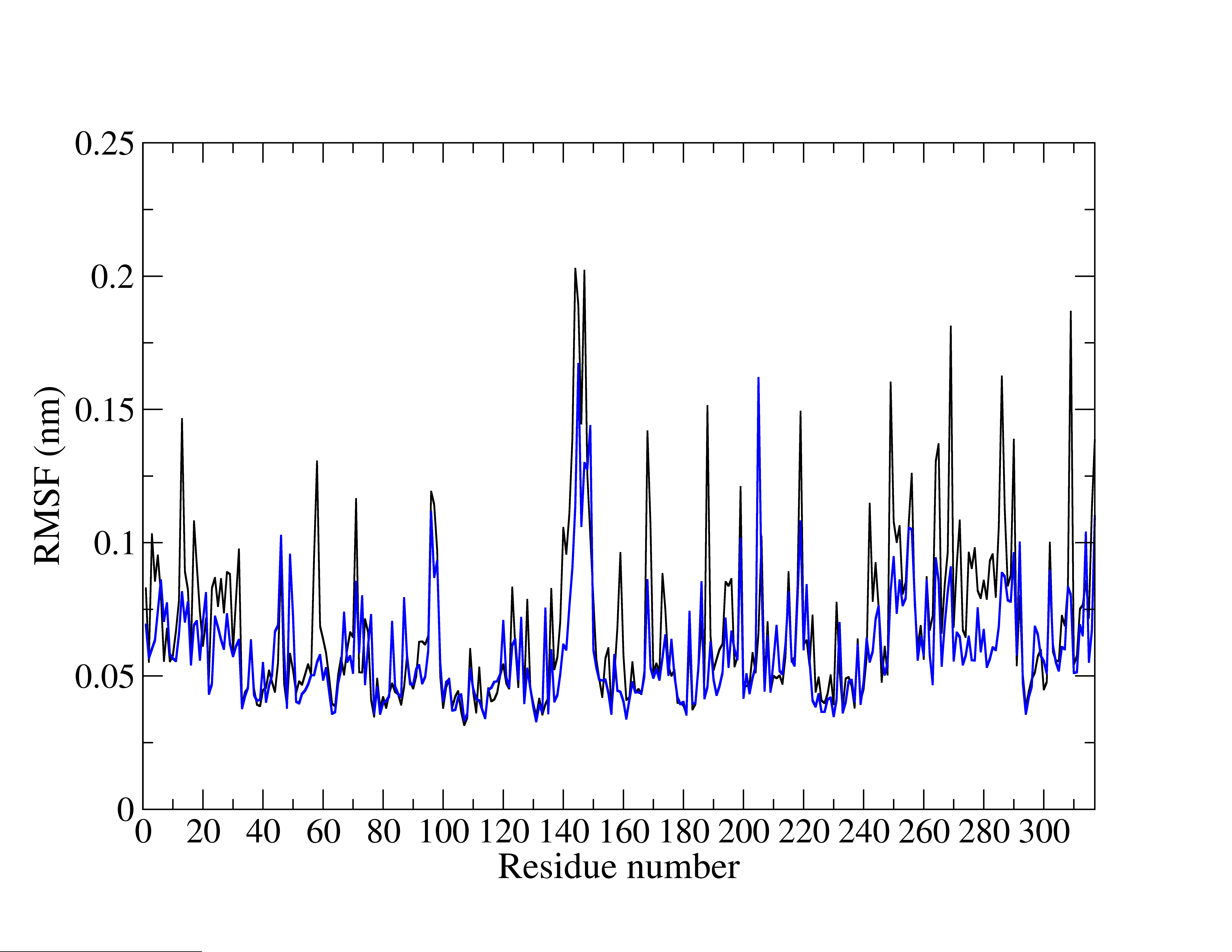

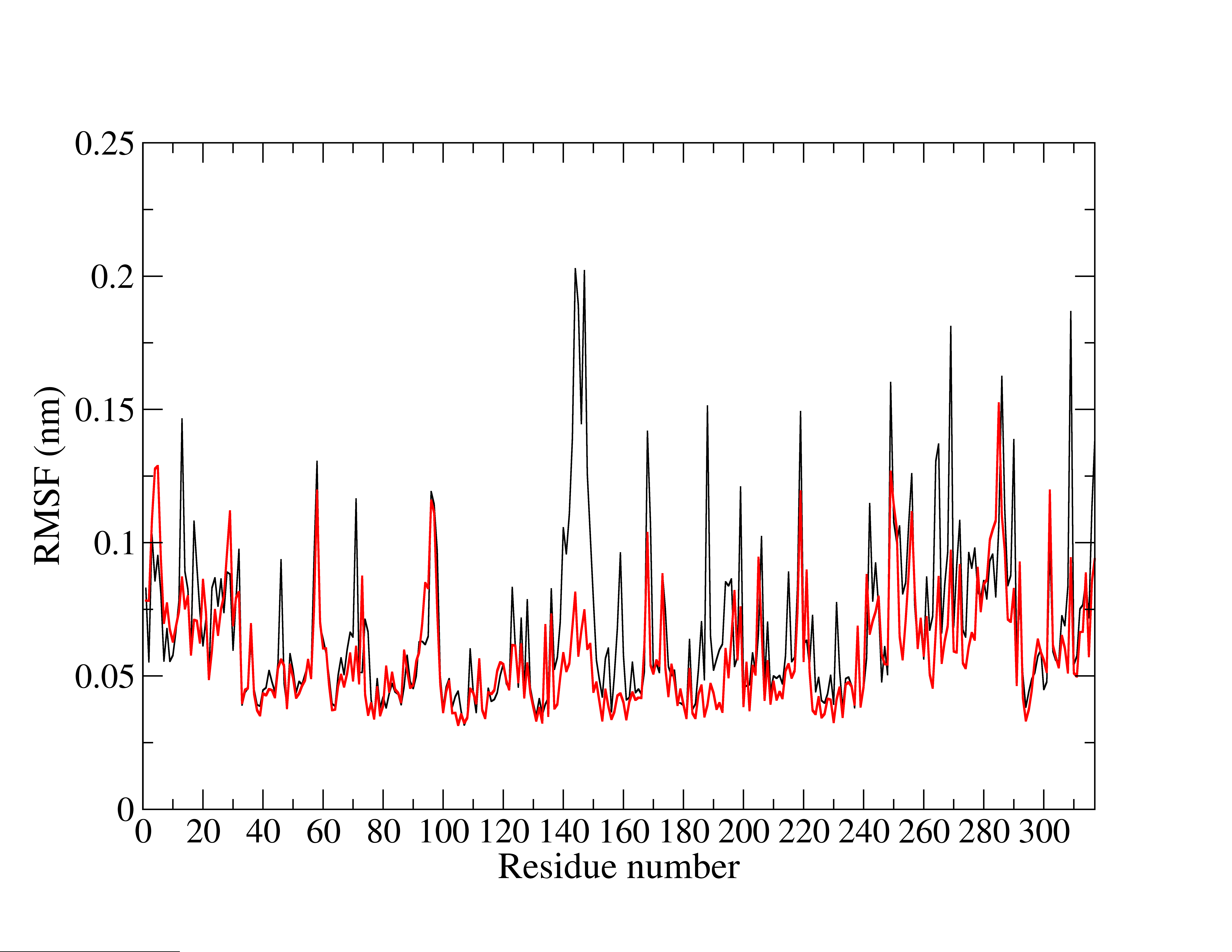


(a)

(b)

*n-*butanol

*tert-*butanol

acetonitrile

cyclohexane

(a)

(b)

Figure S5. Surface area of the CALB in the solvents with (a) OPLSAA forcefield and (b) AMBER forcefield.

Table S1. Average radius of gyration of CALB with OPLSAA forcefield in the solvents studied, calculated from the last structure of the simulations.

| **Solvent** | **Radius of gyration (nm)** |
| --- | --- |
| Crystal structure | 1.805 |
| Water | 1.807 ± 0.004 |
| *n-*butanol | 1.824 ± 0.005 |
| *tert-*butanol | 1.827 ± 0.002 |
| Acetonitrile | 1.840 ± 0.004 |
| Cyclohexane | 1.820 ± 0.002 |

Table S2. Solvent dynamics of *n-*butanol around each α-helix using AMBER forcefield. (Calculated diffusion coefficient for the bulk solvent = 0.310 x 10^-5^ cm^2^/s).

|  | **α1**  **(Ext)** | **α2**  **(Int-Ext)** | **α3**  **(Int-Ext)** | **α4**  **(Int)** | **α5**  **(Ext)** | **α6**  **(Int)** | **α7**  **(Int-Ext)** | **α8**  **(Ext)** | **α9**  **(Int-Ext)** | **α10**  **(Ext)** |
| --- | --- | --- | --- | --- | --- | --- | --- | --- | --- | --- |
| Hydrogen bond lifetime  (ps) | 690  ±35 | 385  ±75 | 560  ±26 | 159  ±49 | 588  ±72 | - | 388  ±69 | 1115  ±111 | 652  ±46 | 345  ±44 |
| Diffusion coefficient (10^-5^ cm^2^/s) | 0.263  ±0.003 | 0.302  ±0.004 | 0.283  ±0.003 | 0.148  ±0.003 | 0.274  ±0.004 | 0.210  ±0.001 | 0.294  ±0.007 | 0.202  ±0.002 | 0.268  ±0.003 | 0.319  ±0.002 |
| Residence time (ps) | 34.0  ±0.9 | 25.8  ±1.0 | 29.8  ± 1.2 | 25.9  ±1.0 | 31.1  ±1.7 | 34.8  ± 1.2 | 27.7  ±1.9 | 35.1  ±1.2 | 33.2  ±0.8 | 22.3  ±0.3 |

**Table S3.** Solvent dynamics of *tert-*butanol around each α-helix using AMBER forcefield. (Calculated diffusion coefficient for the bulk solvent = 0.196 x 10^-5^ cm^2^/s).

|  | **α1**  **(Ext)** | **α2**  **(Int-Ext)** | **α3**  **(Int-Ext)** | **α4**  **(Int)** | **α5**  **(Ext)** | **α6**  **(Int)** | **α7**  **(Int-Ext)** | **α8**  **(Ext)** | **α9**  **(Int-Ext)** | **α10**  **(Ext)** |
| --- | --- | --- | --- | --- | --- | --- | --- | --- | --- | --- |
| Hydrogen bond lifetime  (ps) | 748  ±34 | 659  ±76 | 320  ±43 | - | 339  ±67 | - | 773  ±30 | 249  ±39 | 792  ±92 | 115  ±59 |
| Diffusion coefficient (10^-5^ cm^2^/s) | 0.176  ±0.002 | 0.140  ±0.004 | 0.185  ±0.006 | 0.167  ±0.006 | 0.178  ±0.003 | 0.156  ±0.006 | 0.130  ±0.006 | 0.178  ±0.008 | 0.120  ±0.004 | 0.196  ±0.004 |
| Residence time (ps) | 41.2  ±1.5 | 38.2  ±1.4 | 32.0  ±1.3 | 15.6  ±1.7 | 33.4  ±1.2 | 19.7  ±1.9 | 44.0  ±1.5 | 10.3  ±1.6 | 40.1  ±1.4 | 22.3  ±0.3 |

Table S4. Solvent dynamics of acetonitrile around each α-helix using AMBER forcefield. (Calculated diffusion coefficient for the bulk solvent = 2.30 x 10^-5^ cm^2^/s)

|  | **α1**  **(Ext)** | **α2**  **(Int-Ext)** | **α3**  **(Int-Ext)** | **α4**  **(Int)** | **α5**  **(Ext)** | **α6**  **(Int)** | **α7**  **(Int-Ext)** | **α8**  **(Ext)** | **α9**  **(Int-Ext)** | **α10**  **(Ext)** |
| --- | --- | --- | --- | --- | --- | --- | --- | --- | --- | --- |
| Hydrogen bond lifetime  (ps) | 35.8  ±2.6 | 37.2  ±1.0 | 53.7  ±10.2 | 840  ±125 | - | - | 20.0  ±2.0 | - | 40.2  ±3.5 | 31.8  ±10.7 |
| Diffusion coefficient  (10^-5^ cm^2^/s) | 1.48  ±0.04 | 1.26  ±0.05 | 1.04  ±0.05 | 0.67  ±0.05 | 1.55  ±0.04 | 0.86  ±0.05 | 1.54  ±0.06 | 1.53  ±0.04 | 0.88  ±0.01 | 1.52  ±0.04 |
| Residence time (ps) | 40.2  ±0.5 | 45.6  ±0.7 | 42.0  ±0.9 | 19.0  ±1.1 | 32.9  ±0.6 | 19.7  ±1.9 | 29.4  ±1.1 | 39.6  ±0.9 | 47.6  ±0.5 | 40.0  ±0.3 |

**Table S5.** Solvation dynamics of cyclohexane around each α-helix using AMBER forcefield. (Calculated diffusion coefficient for the bulk solvent = 0.95 x 10^-5^ cm^2^/s).

|  | **α1**  **(Ext)** | **α2**  **(Int-Ext)** | **α3**  **(Int-Ext)** | **α4**  **(Int)** | **α5**  **(Ext)** | **α6**  **(Int)** | **α7**  **(Int-Ext)** | **α8**  **(Ext)** | **α9**  **(Int-Ext)** | **α10**  **(Ext)** |
| --- | --- | --- | --- | --- | --- | --- | --- | --- | --- | --- |
| Diffusion coefficient  (10^-5^ cm^2^/s) | 0.75  ±0.02 | 0.68  ±0.01 | 0.67  ±0.02 | 0.64  ±0.02 | 0.75  ±0.02 | 0.66  ±0.02 | 0.74  ±0.01 | 0.77  ±0.02 | 0.64  ±0.01 | 0.94  ±0.02 |
| Residence time (ps) | 29.1  ±1.0 | 29.1  ±0.9 | 31.5  ±1.0 | 36.9  ±1.1 | 25.6  ±0.9 | 38.1  ±1.4 | 29.1  ±0.5 | 13.6  ±0.3 | 33.7  ±0.6 | 10.8  ±0.6 |
